# A bipotential organoid model of respiratory epithelium recapitulates high infectivity of SARS-CoV-2 Omicron variant

**DOI:** 10.1038/s41421-022-00422-1

**Published:** 2022-06-17

**Authors:** Man Chun Chiu, Cun Li, Xiaojuan Liu, Yifei Yu, Jingjing Huang, Zhixin Wan, Ding Xiao, Hin Chu, Jian-Piao Cai, Biao Zhou, Ko-Yung Sit, Wing-Kuk Au, Kenneth Kak-Yuen Wong, Gang Li, Jasper Fuk-Woo Chan, Kelvin Kai-Wang To, Zhiwei Chen, Shibo Jiang, Hans Clevers, Kwok Yung Yuen, Jie Zhou

**Affiliations:** 1 Department of Microbiology, School of Clinical Medicine, Li Ka Shing Faculty of Medicine, The University of Hong Kong, Pokfulam, Hong Kong, China; 2 State Key Laboratory of Emerging Infectious Diseases, The University of Hong Kong, Hong Kong, China; 3 Centre for Virology, Vaccinology and Therapeutics, Hong Kong Science and Technology Park, Hong Kong, China; 4 AIDS Institute, Li Ka Shing Faculty of Medicine, The University of Hong Kong, Hong Kong, China; 5 Department of Surgery, Li Ka Shing Faculty of Medicine, The University of Hong Kong, and Queen Mary Hospital, Hong Kong, China; 6 Department of Otolaryngology-Head and Neck Surgery, Precision Medicine Center, Nanfang Hospital, Southern Medical University, Guangzhou, Guang dong China; 7 Carol Yu Centre for Infection, The University of Hong Kong, Pokfulam, Hong Kong China; 8 Key Laboratory of Medical Molecular Virology (MOE/NHC/CAMS), Institute of Infectious Disease and Biosecurity, School of Basic Medical Sciences, Fudan University, Shanghai, China; 9 Oncode Institute, Hubrecht Institute, Royal Netherlands Academy of Arts and Sciences (KNAW), and University Medical Center (UMC) Utrecht, Utrecht, the Netherlands

**Keywords:** Adult stem cells, Stem-cell differentiation

## Abstract

The airways and alveoli of the human respiratory tract are lined by two distinct types of epithelium, which are the primary targets of respiratory viruses. We previously established long-term expanding human lung epithelial organoids from lung tissues and developed a ‘proximal’ differentiation protocol to generate mucociliary airway organoids. However, a respiratory organoid system with bipotential of the airway and alveolar differentiation remains elusive. Here we defined a ‘distal’ differentiation approach to generate alveolar organoids from the same source for the derivation of airway organoids. The alveolar organoids consisting of type I and type II alveolar epithelial cells (AT1 and AT2, respectively) functionally simulate the alveolar epithelium. AT2 cells maintained in lung organoids serve as progenitor cells from which alveolar organoids derive. Moreover, alveolar organoids sustain a productive SARS-CoV-2 infection, albeit a lower replicative fitness was observed compared to that in airway organoids. We further optimized 2-dimensional (2D) airway organoids. Upon differentiation under a slightly acidic pH, the 2D airway organoids exhibit enhanced viral replication, representing an optimal in vitro correlate of respiratory epithelium for modeling the high infectivity of SARS-CoV-2. Notably, the higher infectivity and replicative fitness of the Omicron variant than an ancestral strain were accurately recapitulated in these optimized airway organoids. In conclusion, we have established a bipotential organoid culture system able to reproducibly expand the entire human respiratory epithelium in vitro for modeling respiratory diseases, including COVID-19.

## Introduction

The COVID-19 pandemic caused by SARS-CoV-2 has posed an unprecedented threat to public health globally^1^. SARS-CoV-2 has evolved constantly since late 2020. The recently emerged Omicron variant (B.1.1.529) surged quickly and produced a tsunami of COVID-19 cases worldwide. Compelling evidence indicated its notably increased transmission rate^2^. COVID-19 patients develop a broad spectrum of symptoms, ranging from mild upper respiratory illness to fatal pneumonia^3,4^. The respiratory epithelium, particularly the airway epithelium, is the primary infection site of SARS-CoV-2. Yet, viral pneumonia suggests that alveoli in the distal respiratory tract are also susceptible to the virus. Indeed, airway ciliated cells and alveolar epithelial cells are the target cells in COVID-19 patients and SARS-CoV-2-infected nonhuman primates^5–8^.

The human respiratory tract is lined with two distinct types of epithelium, i.e., airway and alveolar epithelium. The former lines the airways from the nasal cavity (except nasal vestibule) to the terminal bronchiole, and consists of four major types of epithelial cells, i.e., ciliated, goblet, club, and basal cells. The human alveolar sac, the basic unit of oxygen exchange, is lined with alveolar epithelium, which is composed of flat type I alveolar epithelial (AT1) cells and cuboidal type II alveolar epithelial (AT2) cells. AT1 cells are involved in gas exchange, while AT2 cells synthesize, secrete, and recycle surfactants to regulate the alveolar surface tension and maintain alveolar stability. Currently, immortalized cell lines such as A549 or Calu3 are commonly utilized to study respiratory viruses, including SARS-CoV-2. However, these homogeneous cell lines are unable to simulate the multicellular complexity and functional diversity of human respiratory epithelia, let alone model respiratory infections. Thus, the in vitro cultivated primary airway and alveolar epithelial cells are harnessed to delineate SARS-CoV-2 infection^9–11^. However, the limited proliferative capacity of primary cells^12^ in vitro restricts their utility in routine experimentation for in-depth characterization of SARS-CoV-2 infection.

Organoids derived from pluripotent stem cells (PSCs), such as embryonic stem cells and induced pluripotent stem cells, as well as organ-specific adult stem cells (ASCs), can simulate key structural and functional properties of tissues of interest^13^. SARS-CoV-2 infection was reported in PSC-derived lung organoids^14,15^. Some disadvantages of these approaches, however, include the fetal-like properties that appear to be inherent to PSC-derived organoids, and the complicated manipulation required to generate PSC-derived organoids. Alveolar spheres or AT2 organoids generated from purified primary AT2 cells^16,17^, and bronchioalveolar organoids derived from fetal lung bud tips^18^ were used to study SARS-CoV-2 lung diseases and revealed SARS-CoV-2 lung infection. These organoids are superior to existing in vitro models of cell lines and primary cells in terms of reproducibility and biological relevance. Yet, a respiratory organoid system with bipotential differentiation of the airway and alveolar epithelium remains elusive.

We have previously established human lung organoids directly from ASCs in normal lung tissues with a high success rate of over 90%. These lung organoids, consisting exclusively of epithelial cells, are stably expanded in the expansion medium for at least one year^19–21^. Since the dominant cell population in the airway epithelium, ciliated cells, were underrepresented in the lung organoids, we developed a proximal differentiation protocol and established three-dimensional (3D) and 2D airway organoids that morphologically and functionally simulate the human airway epithelium to a near-physiological level^19^. In this feeder-free culture system, the expansion medium enables initial derivation and long-term expansion of lung organoids by directing the organoids toward an immature state, while the proximal differentiation protocol generates epithelial domains faithfully phenocopying the native airway epithelium. These physiologically-active airway organoids have been used in various studies^22,23^, including modeling human airway epithelial cell interaction with viruses. We demonstrated that these differentiated airway organoids adequately reproduced the differential infectivity of influenza virus strains as manifested in virus surveillance^19^.

The present study started from the observation that expanding lung organoids also contain club, basal/basal-like and AT2 cells, all reported as the facultative progenitors of AT2 and AT1 cells^24–26^. This prompted us to attempt distal differentiation of lung organoids and generate alveolar organoids consisting of AT1 and AT2 cells. Currently, we generally rely on epidemiological and animal studies to define the infectivity of SARS-CoV-2 variants. However epidemiological observations are readily confounded by control measures or founder effect, SARS-CoV-2 animal models involve issues such as virus adaptation and species variation^27^. Hence, a readily available in vitro model is urgently needed to elucidate virus-host interaction within human respiratory epithelial cells, the primary target of respiratory viruses. In this connection, we sought to establish a physiologically-active in vitro model of human respiratory epithelium for modeling the high infectivity of SARS-CoV-2 and assessing the infectivity of SARS-CoV-2 emerging variants.

## Results

### Derivation and characterization of alveolar organoids

Human lung organoids were established from lung tissues of multiple donors who underwent lung resection due to disease conditions. These lung organoids were found to be stably expanded for at least one year in the expansion medium without any feeder or stromal cells. Key growth factors in the expansion medium include R-spondin, Noggin, FGF7, and FGF10. Surprisingly, apart from the airway cell types described previously^19^, we found a substantial number of AT2 cells in expanding lung organoids after testing several AT2 markers and antibodies. Flow cytometry analysis showed 20%–30% SFTPC^+^ AT2 cells, as well as 3%–10% AQP5^+^ AT1 cells (Supplementary Fig. S1a, b). Consistent with the previous observation, club cells were the predominant cell type among the cells of airway lineage. Overall, although expression levels of cell-type markers varied (Supplementary Fig. S2), epithelial cells of the airway and alveolar lineages were maintained in the lung organoids during long-term expansion.

**Fig. 1:**
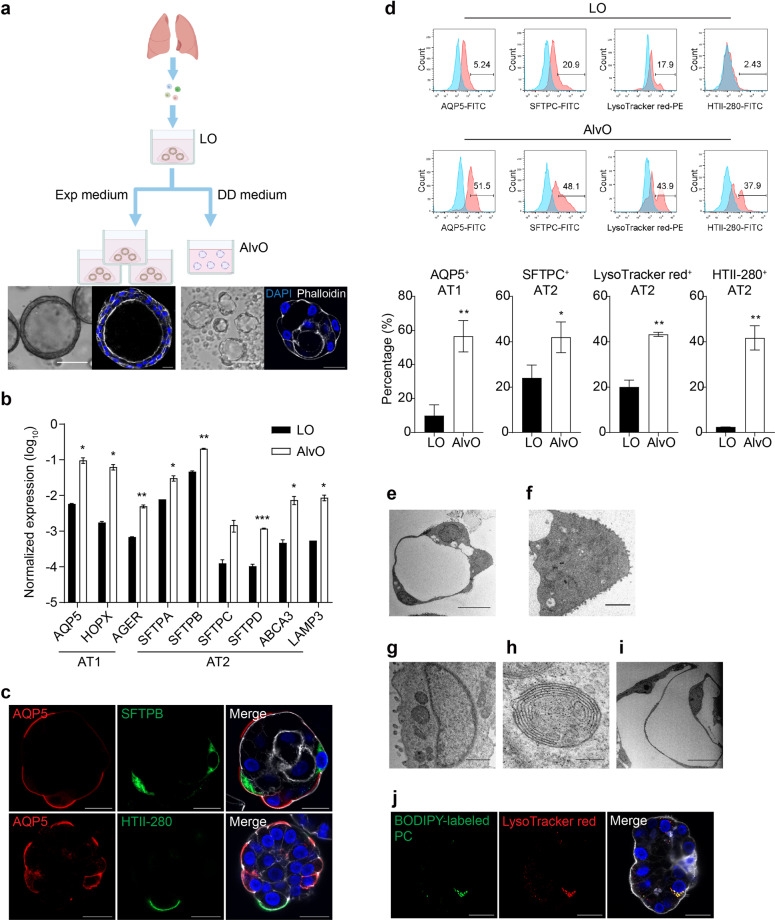
Characterization of differentiated alveolar organoids. **a** A schematic graph outlines the generation of alveolar organoids. Photomicrographs display live lung organoids (LO) and differentiated alveolar organoids (AlvO); scale bar, 100 µm. Confocal images present LO and AlvO labeled with DAPI (blue) and Phalloidin (white); scale bar, 20 µm. See also Supplementary Figs. S1 and S2. **b** Parental LOs and the differentiated AlvOs were assessed for the expression level of AT1- and AT2-cell marker genes. Data represent means ± SD of a representative experiment in one organoid line, *n* = 2. Two-tailed unpaired Student’s *t*-test. See also Supplementary Figs. S3–S6. **c** AlvOs were subjected to immunofluorescence staining to label AQP5^+^ AT1 cells (red), SFTPB^+^ (green, top), or HTII-280^+^ (green, bottom) AT2 cells. Nuclei and actin filaments were counterstained with DAPI (blue) and Phalloidin-647 (white), respectively. Scale bar, 20 µm. **d** Parental LOs and differentiated AlvOs were applied to flow cytometry to examine the abundance of AQP5^+^ AT1 and SFTPC^+^/LysoTracker red^+^/HTII-280^+^ AT2 cells. Representative histograms are shown on the top. Red, cells stained with specific antibodies or dyes; blue, cells stained with isotype controls or mock-stained. Data underneath represent means ± SD of a representative experiment in one organoid line, *n* = 3 for AQP5 and SFTPC, *n* = 2 for LysoTracker red and HTII-280. Two-tailed unpaired Student’s *t*-test. See also Supplementary Fig. S7. **e**–**i** AlvOs were examined with transmission electron microscopy. **e** Cuboidal AT2 cells and thin AT1 cells in an alveolar organoid. Scale bar, 10 µm. **f** Microvilli on an AT2 cell. Scale bar, 2 µm. **g** Lamellar bodies in an AT2 cell. Scale bar, 1 µm. **h** A lamellar body within an AT2 cell. Scale bar, 200 nm. **i** An AT1 cell with thin cytoplasm. Scale bar, 10 µm. **j** AlvOs preincubated with BODIPY phosphatidylcholine (green) were further labeled with LysoTracker red (red) and then subjected to live-cell confocal imaging. Organoids were counterstained with Hoechst (blue) and CellMask™ plasma membrane stain (white). Scale bar, 20 µm.

Prior studies indicated that club, basal/basal-like, and AT2 cells were the facultative progenitors of AT2 and AT1 cells^24–26^. This prompted us to induce lung organoid differentiation into alveolar organoids enriched for mature AT1 and AT2 cells. After screening an array of growth factors for the propensity to induce alveolar differentiation, we defined a distal differentiation protocol. Briefly, lung organoids were enzymatically digested into single cells, and then suspension-cultured in the distal differentiation (DD) medium composed of Wnt3a conditioned medium and alveolar maturation additives: dexamethasone, 8-bromo-cyclic AMP, and 3-isobutyl-1-methylxanthine^28,29^. After a 2-week incubation, single cells from the lung organoids grew into cystic clusters composed of cuboidal and thin cells (Fig. 1a), a morphology reminiscent of alveoli. When the single cells were, instead, embedded in Matrigel overlaid with the expansion (Exp) medium, they proliferated and reformed the original expanding lung organoids with the typical morphology of thick walls and a central lumen (Fig. 1a). This was conceivable since enzymatic digestion of lung organoids into single cells followed by 3D culture in Matrigel with the expansion medium is one of the approaches we routinely split and passage lung organoids.

Compared to lung organoids of the same line in the expansion culture, the organoids subjected to the distal differentiation protocol displayed significantly upregulated canonical AT1 cell markers (AQP5, HOPX, and AGER) and AT2 cell markers (SFTPA, SFTPB, SFTPC, SFTPD, ABCA3, and LAMP3) (Fig. 1b). Consistent and significant upregulation of these cell-type markers was reproduced in lung organoids from at least two other donors upon distal differentiation (Supplementary Fig. S3a, b). Moreover, lung organoids had to be suspension-cultured in non-attachment plates to achieve alveolar differentiation rather than 3D embedded in Matrigel. The organoids in Matrigel with DD medium failed to achieve the same level of differentiation since these organoids were prone to assume a morphology of thick-wall rather than cystic (Supplementary Fig. S4a) and they invariably exhibited a decreased SFTPC expression (Supplementary Fig. S4b). In line with the essential role of Wnt signaling for alveolar differentiation^30^, the addition of a Wnt agonist CHIR99021 promoted the expression level of AT1 and AT2 marker genes to a level comparable to that of the Wnt3a conditioned medium (Supplementary Fig. S5). To verify the reproducibility of the protocol, we performed distal differentiation in the lung organoids from one donor during six consecutive passages and measured the transcripts of AT1 and AT2 cell markers pairwise in undifferentiated lung organoids of each passage and the derived alveolar organoids. During the six consecutive passages, the transcripts of these cell-type markers in undifferentiated lung organoids fluctuated, albeit persisted, consistent with the results shown in Supplementary Fig. S2. Nonetheless, the distal differentiation protocol enabled robust and consistent alveolar differentiation (Supplementary Fig. S6). The results also corroborated the stability of lung organoids as a self-renewable source to differentiate into alveolar organoids.

Abundant AQP5^+^ AT1 and SFTPB^+^ or HTII-280^+^ AT2 cells were discernible in the alveolar organoids by immunofluorescence staining (Fig. 1c). Consistent with a recent report of human intestinal organoids^31^, the alveolar organoids in suspension culture exhibited an apical-out polarity, which is revealed by the exterior localization of AQP5 and HTII-280, the proteins intrinsically expressed on the apical membrane of AT1 and AT2 cells, respectively. Flow cytometry analysis demonstrated that AQP5^+^ AT1 cell, and AT2 cell labeled with SFTPC, HTII-280, and LysoTracker red, were significantly enriched in alveolar organoids compared to those in expanding lung organoids. The percentages of both AT1 and AT2 cells increased to around 50% in the alveolar organoids (Fig. 1d). The abundance of AQP5 protein also increased remarkably after distal differentiation as determined by the mean fluorescence intensity of AQP5^+^ cells (Supplementary Fig. S7). Transmission electron microscopy confirmed that alveolar organoids were composed of AT1 and AT2 cells with the typical micromorphology (Fig. 1e). AT2 cells feature microvilli (Fig. 1f) and cytoplasmic lamellar bodies (Fig. 1g, h), a secretory vesicle containing surfactant proteins. AT1 cells in the organoids exhibited a thin and flat morphology (Fig. 1i), a characteristic of AT1 cells in vivo.

We next assessed the functionality of alveolar organoids by examining the capacity of AT2 cells to absorb surfactant and phospholipids, an essential physiological function of native AT2 cells in vivo^29,32^. To this end, the alveolar organoids were preincubated in the DD medium supplemented with a green fluorescent-labeled phosphocholine, β-BODIPY FL C12-HPC, or were mock-treated with the DD medium only. LysoTracker red, a fluorescent dye that preferentially binds acidic organelles, can label lamellar bodies and is commonly used for live-cell imaging and cell sorting of AT2 cells^28,32^. After further incubation with LysoTracker red, we found colocalization of the green fluorescent phosphocholine with LysoTracker red-labeled lamellar bodies within the AT2 cells in alveolar organoids by live-cell confocal imaging (Fig. 1j). In contrast, no green fluorescent particles were discernible in LysoTracker red-labeled AT2 cells within the mock-treated alveolar organoids (results not shown). The results indicate that AT2 cells in the alveolar organoids were functional and capable of uptake surfactant components.

### Identification of AT2 cell as the progenitor for alveolar organoids

**Fig. 2:**
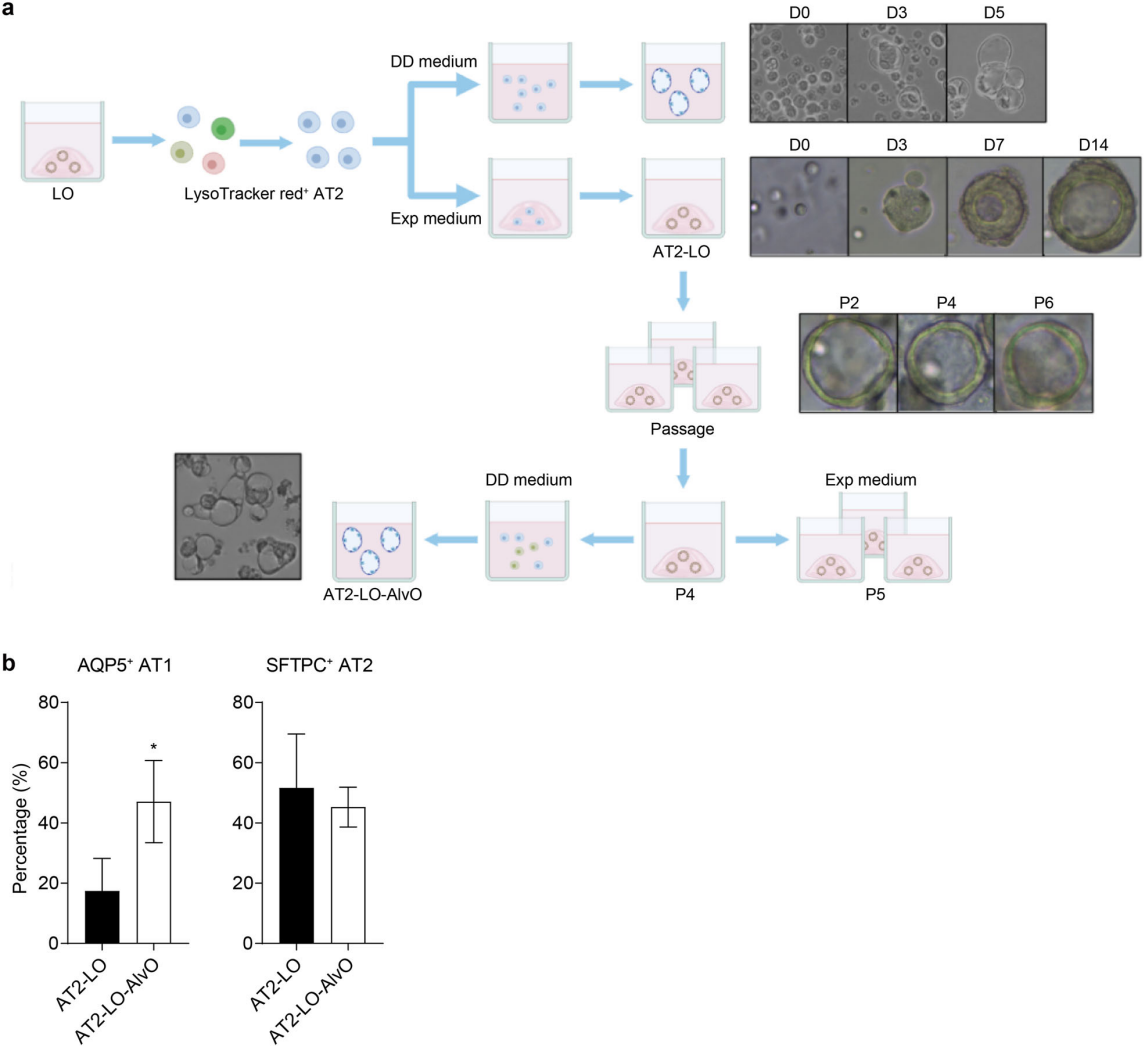
Identification of AT2 cells as progenitor cells to derive alveolar organoids. **a** A schematic graph outlines the experimental procedure. Purified AT2 cells undergoing DD protocol developed cystic structures similar to AlvOs. Photomicrographs on day 0 (D0), day 3, and day 5 (magnification 200×) are shown. Single AT2 cells 3D incubated in the expansion medium assumed a morphology similar to lung organoid (AT2-LO) during the 2-week culture. Photomicrographs show a growing organoid from day 0 to day 14 (magnification 100×). AT2-LOs were consecutively passaged for 6 months. Photomicrographs show the organoids of a second (P2), fourth (P4), and sixth (P6) passage (magnification 100×). AT2-LOs of the fourth passage subjected to DD protocol formed cystic AT2-LO-AlvOs on day 4 (magnification 200×). **b** AT2-LOs of the fourth passage (*n* = 4) and the derived AT2-LO-AlvOs (*n* = 2) were applied to flow cytometry to examine the abundance of AT1 and AT2 cells. Data represent means ± SD of a representative experiment. Two-tailed unpaired Student’s *t*-test. See also Supplementary Fig. S8.

AT2 cells are facultative stem cells that maintain alveolar epithelium in mouse and human lungs during physiological homeostasis and after injury^17,24,33^. Thus, we reasoned that AT2 cells in lung organoids might be the progenitor cells from which alveolar organoids derive. To test the hypothesis, we sorted AT2 cells from lung organoids with LysoTracker red and then embedded these AT2 cells in Matrigel overlaid with the expansion medium, or incubated these cells in the DD medium as suspension culture (Fig. 2a). An α-HTII-280 against apical membrane protein on mature AT2 cells is commonly used to purify native AT2 cells from human lung tissues^16,17^. However, the attempt to purify AT2 cells with α-HTII-280 failed since it labeled less than 3% of cells in lung organoids, consistent with the result shown in Fig. 1d, whereas LysoTracker red-positive cells accounted for 15%–30% in the same organoids (Supplementary Fig. S8a). The results indicate that the LysoTracker red-positive “AT2 cells” in expanding lung organoids might not be sufficiently mature compared to native AT2 cells in human lung tissues. From day 4 or 5, the purified single AT2 cells undergoing distal differentiation developed an alveolar-like morphology, similar to the alveolar organoids directly derived from lung organoids with the aforementioned distal differentiation protocol. In contrast, the AT2 cells subjected to 3D culture with the expansion medium underwent a clonal expansion and formed organoids resembling lung organoids with thick walls and a central lumen. In addition, these single AT2 cell-derived lung organoids (AT2-LOs) were continuously expanded for 6 months (Fig. 2a). The AT2-LOs were mainly composed of AT2 and AT1 cells, with a minor population of club cells (Supplementary Fig. S8b)

We then tested whether the expanding AT2-LOs could differentiate into AT2-LO-derived alveolar organoids (AT2-LO-AlvOs) upon distal differentiation. To this end, a proportion of AT2-LOs at the fourth passage were applied to distal differentiation, while the others underwent further expansion. On day 4 after distal differentiation, the organoids assumed the morphology of cystic alveolar organoids (Fig. 2a). We next examined the cellular composition of the parental AT2-LOs of the fourth passage and the derived AT2-LO-AlvOs by flow cytometry. AT2-LOs contained around 20% AT1 cell and 50% AT2 cell, whereas AT2-LO-AlvOs contained ~50% of each cell type (Fig. 2b). Thus, AT1 cells became significantly enriched in AT2-LO-AlvOs than the original AT2-LOs. Importantly, AT2-LO-AlvOs derived from expanding AT2-LOs exhibited a cell composition comparable to that of alveolar organoids directly derived from lung organoids (Fig. 1d). Collectively, our results indicate that the immature AT2 cells maintain a clonogenic capacity in the expanding lung organoids, thus serving as alveolar progenitor cells to generate alveolar organoids upon distal differentiation.

### Characterization of alveolar and airway organoids derived from expandable lung organoids

**Fig. 3:**
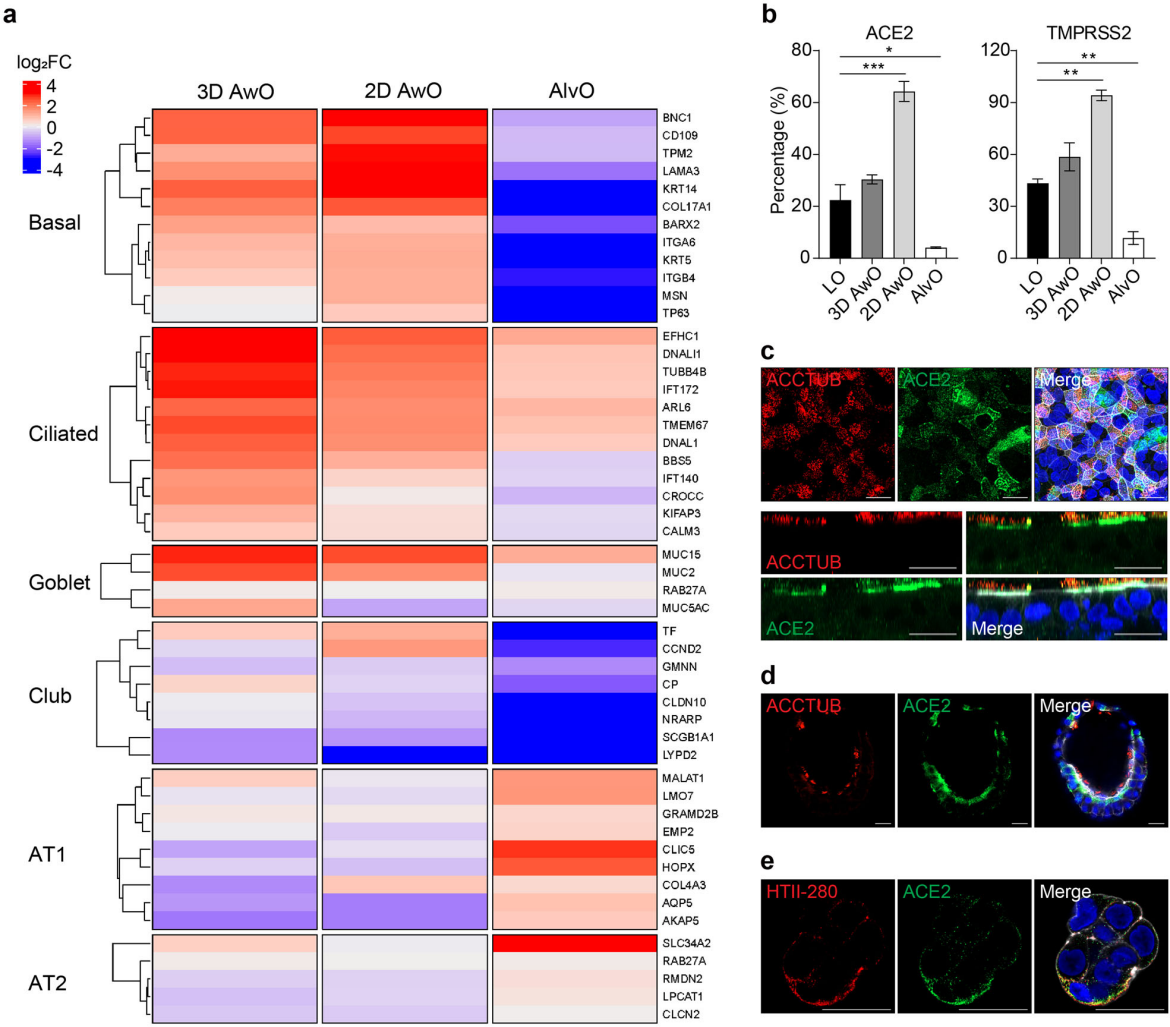
Characterization of alveolar and airway organoids. **a** Lung organoids (LO) were applied to distal or proximal differentiation in parallel to generate alveolar organoids (AlvO) or 3D and 2D airway organoids respectively (3D AwO, 2D AwO). Heatmap depicts the top differentially expressed cell markers in the differentiated airway and alveolar organoids relative to undifferentiated lung organoids. Colored bars represent log_2_ transformed fold change. **b** Expression of ACE2 and TMPRSS2 in LOs and the derived AlvOs, 3D AwOs, and 2D AwOs was examined by flow cytometry. Data represent means ± SD of a representative experiment, *n* = 2. Ordinary one-way ANOVA with Dunnett’s multiple comparison test comparing differentiated organoids to the lung organoids. **c**–**e** Representative confocal images of ACE2 (green) protein. Nuclei and actin filaments were counterstained with DAPI (blue) and Phalloidin-647 (white), respectively. Scale bar, 20 µm. **c** 2D AwOs co-stained with α-ACCTUB (red), en face (top), and cross-section (bottom); **d** 3D AwOs co-stained with α-ACCTUB (red); and **e** an AlvO co-stained with α-HTII-280 (red). See also Supplementary Fig. S9.

We next characterized alveolar organoids compared to the undifferentiated lung organoids and airway organoids derived from the same lung organoids. To this end, expanding lung organoids were induced distal or proximal differentiation in parallel to generate alveolar or airway organoids, respectively, the latter including the 3D airway organoids (3D AwOs) in Matrigel and the 2D airway organoids (2D AwOs) in transwell plates. All organoids were assessed by bulk RNA sequencing (RNA-seq) analysis. Compared to the undifferentiated lung organoids, the airway organoids exhibited increased expression of marker genes of airway lineage, representing ciliated (e.g., EFHC1, DNALI1, and TUBB4B), goblet (e.g., MUC15, MUC2, and MUC5AC), and basal cells (e.g., KRT14, TGFB1, and BNC1); whereas AT1- and AT2-specific genes were generally downregulated (Fig. 3a). In contrast, the alveolar differentiation protocol dramatically and comprehensively boosted AT1- (e.g., CLIC5, HOPX, and AQP5) and AT2-specific genes (e.g., SLC34A2 and GLRX)^34^ while suppressing the marker genes of airway cell types. Thereby, the transcriptomic profile of airway and alveolar organoids relative to lung organoids confirmed airway and alveolar differentiation.

ACE2 is the primary cellular receptor of SARS-CoV-2. In addition, cellular serine proteases, including TMPRSS2, are hijacked to prime coronaviruses for efficient cellular entry^35^. We examined ACE2 and TMPRSS2 expression in lung organoids and the differentiated organoids by flow cytometry analysis. ACE2 expression was the highest in 2D airway organoids, followed by 3D airway organoids (Fig. 3b), whereas alveolar organoids displayed an even lower ACE2 expression than undifferentiated lung organoids. TMPRSS2 showed a similar order of expression in these organoids. Immunofluorescence staining revealed abundant ACE2 distributed mainly on the apical surface of ACCTUB^+^ ciliated cells in 2D airway organoids (Fig. 3c). ACE2 was discernible in ciliated cells in 3D airway organoids (Fig. 3d) and HTII-280^+^ AT2 cells in alveolar organoids (Fig. 3e), yet at lower abundance than 2D airway organoids. Flow cytometry analysis verified that ACCTUB^+^ ciliated cells in airway organoids and SFTPC^+^ AT2 cells in alveolar organoids were the major cell types expressing ACE2 (Supplementary Fig. S9), which is in line with the findings in human respiratory tissues^36^.

### Productive SARS-CoV-2 infection in the airway and alveolar organoids

**Fig. 4:**
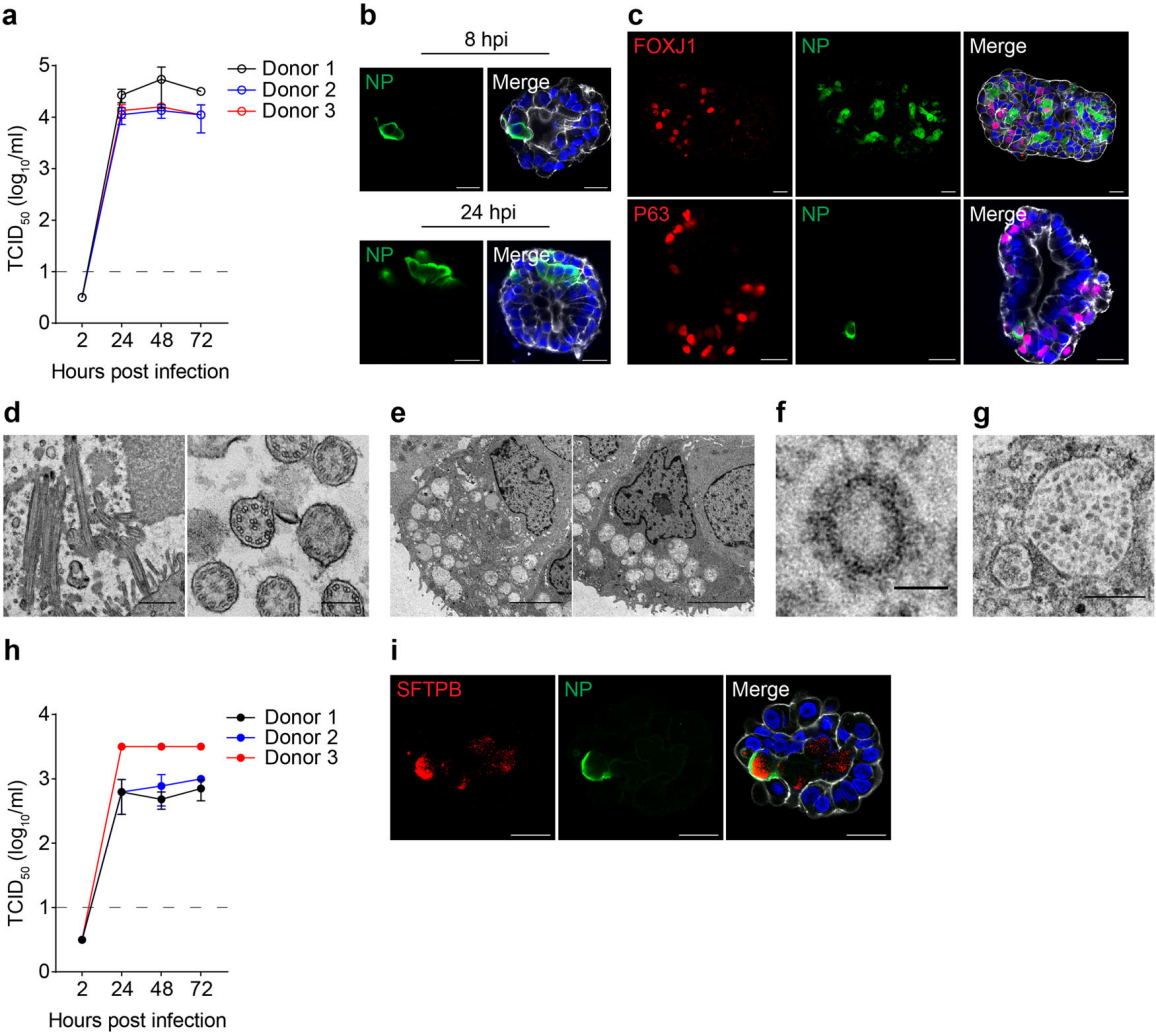
SARS-CoV-2 replication in 3D airway organoids and alveolar organoids. **a** At the indicated hours post-infection, culture media were harvested from three lines of 3D AwOs infected with WT SARS-CoV-2 (MOI = 1) and applied to viral titration by TCID_50_ assay. Data represent means ± SD in three organoid lines, *n* = 3 for each line. The dashed line indicates the detection limit. **b** 3D AwOs inoculated with SARS-CoV-2 (MOI = 2) were fixed at 8 or 24 hpi and immunostained to identify viral nucleoprotein (NP, green) positive cells. Nuclei and actin filaments were counterstained with DAPI (blue) and Phalloidin-647 (white), respectively. Scale bar, 20 µm. **c** After fixation at 24 hpi, SARS-CoV-2-infected AwOs were co-stained with α-NP (green) and α-FOXJ1 (red, top) or α-P63 (red, bottom). Scale bar, 20 µm. **d**–**g** Representative microphotographs of transmission electron microscopy. **d** Ciliated cells. Longitudinal section (left, scale bar 1 µm) and cross-section (right, scale bar 200 nm) of cilia showing microtubules with a 9 + 2 arrangement. **e** Goblet cells. Scale bar, 4 µm. **f** A virion particle in the cytoplasm of SARS-CoV-2-infected (MOI = 5) airway organoids. Scale bar, 50 nm. **g** Virion particles in a secretory vesicle in a cell of infected organoids. Scale bar, 400 nm. **h** At the indicated hours post-infection, culture media were harvested from three lines of AlvOs infected with WT SARS-CoV-2 (MOI = 1) and applied to viral titration by TCID_50_ assay. Data represent means ± SD in three organoid lines, *n* = 3 for each line. **i** Representative confocal images of SARS-CoV-2-infected SFTPB^+^ AT2 cells in an AlvO (MOI = 2). Scale bar, 20 µm.

We examined the infectivity and viral growth of SARS-CoV-2 in 3D human airway organoids derived from lung tissues of three different donors. Wildtype (WT, HKU-001a) SARS-CoV-2 productively infected all three lines of airway organoids. The titer of infectious virus increased up to 3–4 log units at 72 h post-inoculation (hpi) (Fig. 4a). We further verified productive SARS-CoV-2 infection by immunostaining. Individual infected cells were discernible at 8 hpi. At 24 hpi, the virus spread to neighboring cells (Fig. 4b). Besides ciliated cells, basal cells were also targeted by the virus (Fig. 4c), consistent with a recent report of a single-cell RNA sequencing study^37^. We also performed transmission electron microscopy to examine 3D airway organoids and SARS-CoV-2-infected organoids. Many cells in airway organoids displayed the typical cilium with microtubules of 9 + 2 arrangement (Fig. 4d), while others showed the ultrastructure of goblet cells with secretory vesicles in the apical cytoplasm (Fig. 4e), indicating an appreciable mucociliary differentiation of airway organoids as we demonstrated previously^19^. Virion particles with an average size of 70–80 nm (Fig. 4f) and the typical “corona” projections were readily discernible in the cells of virus-inoculated 3D airway organoids (Fig. 4g). We also observed a productive SARS-CoV-2 infection in alveolar organoids from three different donors. As shown in Fig. 4h, viral titer increased significantly after SARS-CoV-2 inoculation. In addition, immunofluorescence staining revealed SFTPB^+^ AT2 cells infected by SARS-CoV-2 (Fig. 4i).

### Optimized 2D airway organoids sustaining more robust SARS-CoV-2 replication

Given the high infectiousness of SARS-CoV-2 and high viral load in respiratory specimens of COVID-19 patients^38^, it appeared to us that SARS-CoV-2 replication in the 3D airway organoids might not be sufficiently robust to recapitulate its high infectivity in the airway epithelial cells of COVID-19 patients. We speculated that the airway organoids might not adequately model the native airway epithelium, leading to compromised SARS-CoV-2 viral growth. The surface liquid in the human airways is slightly acidic, with an average pH of 6.6^39^. It was reported that the low pH of airway surface liquid is one of the key determinants promoting SARS-CoV-2 infection^40^. However, in the 2D airway organoid culture, the proximal differentiation media in both upper and bottom chambers are buffered with HEPES at a pH of 7.4, the physiological pH of interstitial fluid. Thus, we hypothesized that a slightly acidic medium in the top chamber and a physiological alkaline medium in the bottom chamber might better simulate the in vivo milieu and generate more physiologically-active airway organoids.

**Fig. 5:**
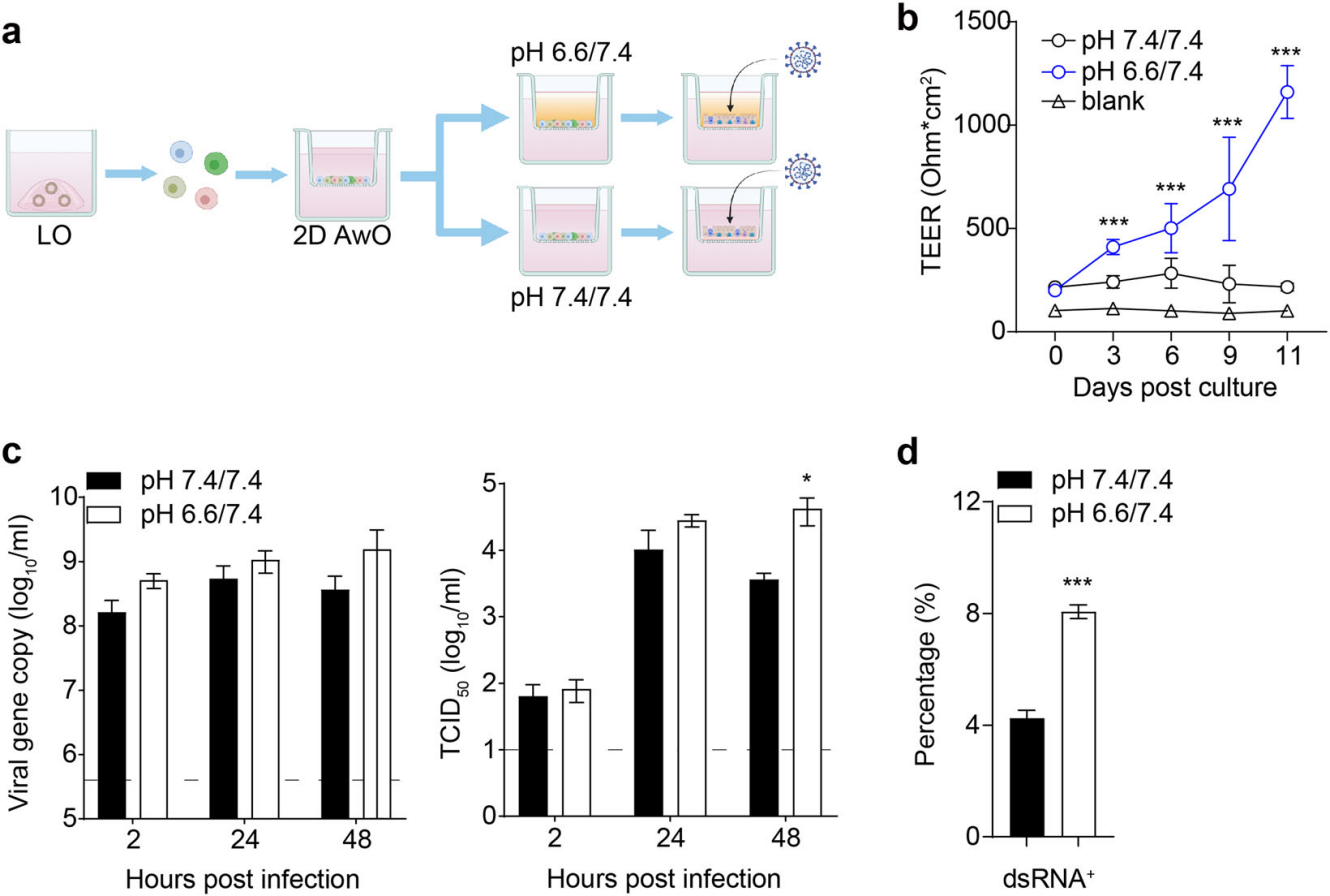
Enhanced SARS-CoV-2 replication in optimized 2D airway organoids. **a** A schematic graph outlines the optimization of 2D airway organoids for modeling SARS-CoV-2 infection. **b** 2D airway organoids incubated with the differentiation medium of pH 6.6/7.4 or 7.4/7.4 were measured for trans-epithelial electrical resistance (TEER) at the indicated days. Data represent means ± SD of a representative experiment, *n* = 9. Two-tailed unpaired Student’s *t*-test. **c** At the indicated hours post-infection with WT SARS-CoV-2 (MOI = 0.1), culture media were harvested from the apical chambers of the 2D airway organoids and applied to viral load detection and viral titration by TCID_50_ assay. Data show means ± SD of a representative experiment, *n* = 3. Two-tailed unpaired Student’s *t*-test. **d** At 24 hpi, cells were harvested from 2D airway organoids infected with SARS-CoV-2 (MOI = 10) and applied to flow cytometry to detect dsRNA^+^ virus-infected cells. Data represent means ± SD of a representative experiment, *n* = 3. Two-tailed unpaired Student’s *t*-test. See also Supplementary Fig. S10.

To this end, after cells reached confluency on the transwells, HEPES in the top medium was changed to a PIPES buffer to create a pH of 6.6, while the original HEPES remained in the bottom medium with a pH of 7.4. Namely, 2D organoids were incubated in the modified differentiation medium with a pH of 6.6/7.4 (top/bottom) or the original medium with a pH of 7.4/7.4 (Fig. 5a). After 12–14 days of proximal differentiation, we noticed an improved cell morphology and increased amounts of beating cilia in organoids at pH 6.6/7.4 compared to those at pH 7.4/7.4 (Supplementary Fig. S10a). Accordingly, the organoids at pH 6.6/7.4 exhibited a significantly higher trans-epithelial electrical resistance (TEER) than those at pH 7.4/7.4 (Fig. 5b), indicating that the optimized organoids formed an enhanced epithelial barrier. Notably, the viral titer was significantly higher in the airway organoids at pH 6.6/7.4 than those at pH 7.4/7.4 (Fig. 5c). At 24 hpi, SARS-CoV-2 infected significantly more cells in the former than in the latter (Fig. 5d). In addition, 2D airway organoids at pH 6.6/7.4 tended to have more ACE2^+^ cells than those at pH 7.4/7.4 (Supplementary Fig. S10b). Collectively, the proximal differentiation medium with a slightly acidic pH in the top chamber improved 2D airway organoids, thus generating a better model of the native airway epithelium. The optimized airway organoids sustained more robust SARS-CoV-2 replication and adequately simulated SARS-CoV-2 high infectivity in the human airways.

### The Omicron variant exhibiting significantly higher infectivity and replicative fitness in 2D airway organoids

**Fig. 6:**
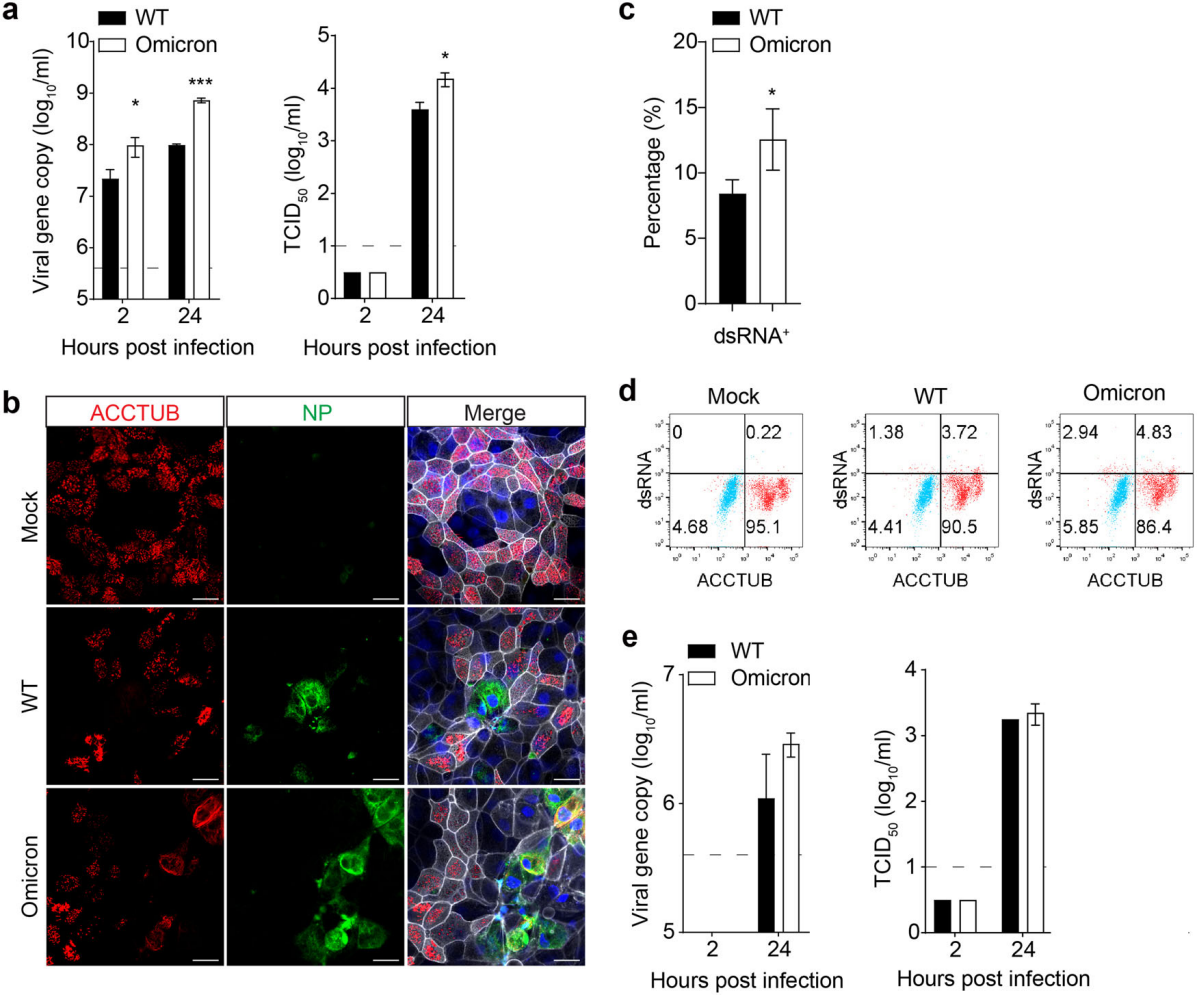
Higher fitness and infectivity of the Omicron variant compared to a WT strain in 2D AwO. **a** At the indicated hours after inoculation with WT or the Omicron variant (MOI = 0.1), culture media were harvested from the apical chambers of the 2D airway organoids and applied to viral load detection and viral titration by TCID_50_ assay. Data show means ± SD of a representative experiment, *n* = 3. Two-tailed unpaired Student’s *t*-test. **b** At 24 hpi (MOI = 2), infected AwOs or mock-infected organoids were co-stained with α-NP (green) and α-ACCTUB (red). Scale bar, 20 µm. **c** At 24 hpi of the Omicron variant and WT virus (MOI = 10), 2D AwOs were dissociated and applied to flow cytometry to detect dsRNA^+^ cells. Data represent means ± SD of a representative experiment, *n* = 4. Two-tailed unpaired Student’s *t*-test. **d** Representative flow cytometry analysis dot plots of mock or infected AwOs (MOI = 1) co-stained with α-dsRNA (PE-Texas red) and α-ACCTUB (FITC). **e** At the indicated hours after inoculation with WT or the Omicron variant (MOI = 1), culture media were harvested from the AlvO and applied to viral load detection and viral titration by TCID_50_ assay. Data show means ± SD of a representative experiment, *n* = 3. See also Supplementary Fig. S11.

Compelling evidence indicates increased transmissibility of the Omicron variant compared to the ancestral strains, which might be attributed to its robust replication in the upper respiratory tract lined with airway epithelium. To collaborate on the higher replicative fitness of the Omicron variant in human airway epithelial cells, we compared the replication kinetics of the Omicron variant and a SARS-CoV-2 ancestral WT strain HKU-001a in the optimized 2D airway organoids. The Omicron variant produced more progeny virions in the culture medium than the WT virus, as shown by a significantly higher viral titer (Fig. 6a). Immunostaining revealed more infected cells in the airway organoids inoculated by the Omicron variant than those inoculated with the WT virus (Fig. 6b). Consistent with the observations in cultured human airway epithelial cells and respiratory cells collected from COVID-19 patients^37,41,42^, we noticed notable cilium damage and loss in the 2D airway organoids infected by both strains (Fig. 6b). At 24 hpi, the Omicron variant infected significantly more cells than the WT virus as quantitatively determined by flow cytometry analysis (Fig. 6c). In addition, the Omicron variant and the WT virus infected both ACCTUB^+^ ciliated cells and ACCTUB− nonciliated cells (Fig. 6d). In contrast, in the 3D alveolar organoids, the WT virus and Omicron variant replicated to a lower titer than that in the 2D airway organoids, and replicative fitness was comparable between these two virus strains (Fig. 6e). However, lower replicative fitness of the Omicron variant was observed in VeroE6/TMPRSS2 cells, which is opposite to the results from organoids and the high transmissibility of the Omicron variant in humans (Supplementary Fig. S11).

## Discussion

We report a two-phase organoid culture system to generate two types of human respiratory epithelium in culture plates. Lung organoids are readily derived from human lung tissues with high efficiency and are expanded stably in the expansion medium for over one year. We induce proximal or distal differentiation in expanding lung organoids to generate airway organoids or alveolar organoids respectively within 2–3 weeks. During the whole procedure, including initial derivation, long-term expansion, and bi-directional differentiation, neither tedious cell purification nor feeder and stromal cells are required. We corroborate the stability of long-term expanding lung organoids, as well as the reproducibility and robustness of the distal differentiation protocol to generate alveolar organoids from lung organoids (Fig. 1 and Supplementary Figs. S1–S6). In addition, the distal differentiation protocol enables the direct generation of alveolar organoids with both AT1 and AT2 cells, unlike other alveolar organoid models in which additional manipulation is required to derive AT1 cells^16,17,43^.

We previously named the organoids derived from lung tissues as “airway organoids” since most organoids display thick walls with variable amounts of ciliated cells. However, subsequent analysis revealed that AT2 cells, albeit insufficiently mature, are present in the “airway organoids” with a percentage of around 20%–30% (Fig. 1d and Supplementary Fig. S1). This prompted us to change to a more appropriate term, lung organoids. The presence of abundant “AT2 cells” in lung organoids is conceivable since R-spondin 1, an agonist of Wnt signaling was one of the essential components of the lung organoid expansion medium. Previous studies indicated that canonical Wnt signaling enables long-term AT2 cell expansion^17,33,44^. However, in terms of proportion and maturation state, “AT2 cells” maintained in the lung organoids are not comparable to native AT2 counterparts in the human alveolar epithelium since the “AT2 cells” in lung organoids were minimally immunoreactive to the α-HTII-280 (Fig. 1d and Supplementary Fig. S8), an antibody recognizing an apical membrane protein on mature AT2 cells^45^.

Prior studies suggested that Wnt signaling drove AT2 cell proliferation, differentiation, and AT2 to AT1 trans-differentiation^30,46,47^. Hence, we attempted distal differentiation in lung organoids and generated alveolar organoids consisting of AT1 and functional AT2 cells (Fig. 1 and Supplementary Fig. S3). The AT2 cells in alveolar organoids are comparably labeled by the α-HTII-280 and α-SFTPC with a remarkably increased abundance to around 50% (Fig. 1d), approximate to the average proportion of AT2 cells in the native alveolar epithelium^48^. Furthermore, electron microscopy reveals abundant cytosolic lamellar bodies and microvilli, which are the characteristic ultrastructures of native AT2 cells (Fig. 1e–h). Notably, live-cell imaging recorded these AT2 cell uptake of surfactant components, indicating active functionality akin to their native counterparts (Fig. 1j). In addition, the AT1 cells in the alveolar organoids are immunoreactive to an α-AQP5 that specifically recognizes native AT1 cells^49^ (Fig. 1d) and display a typically thin and slim morphology under an electron microscope, similar to the native AT1 cells within the alveolar sacs (Fig. 1i). We also displayed AT1 and AT2 cells in the alveolar organoids after immunofluorescence labeling of the cell-type markers (Fig. 1c). However, the AT1 cells appeared cuboid, which may be an artifact as a result of repeated staining and washing manipulations during the immunolabeling. The brightfield images of live alveolar organoids also showed cells with very thin morphology (Fig. 1a).

Similar to ASC-derived human intestinal organoids^50^, the undifferentiated lung organoids maintained in the expansion medium with R-spondin and Noggin retained the clonogenicity of progenitor cells. The expansion medium sustained the long-term expansion of lung organoids by directing the organoids toward an immature state. Unlike Lgr5^+^ as the adult stem cell in undifferentiated intestinal organoids^51^, expanding lung organoids harbor airway and alveolar progenitor cells, as well as differentiated or intermediately-differentiated cell populations of both lineages (Supplementary Fig. S1). We defined the proximal^19^ and distal differentiation conditions that selectively stimulate the airway and alveolar progenitor cells to generate airway and alveolar organoids, respectively. Afterward, we moved forward to identify progenitor cells in the lung organoids that enabled the bi-directional differentiation. Here we demonstrate that immature AT2 cells in the expanding lung organoids are the progenitor cell for alveolar differentiation (Fig. 2). While we conducted experiments to identify the progenitor cell in lung organoids for airway differentiation, a “human distal lung organoid” was reported recently^43^. Similar to our undifferentiated lung organoids, these distal lung organoids, also derived from peripheral lung tissues, consisted of a mixed population of cystic AT2 organoids and solid KRT5^+^ basal organoids. AT2 cells in the culture were the progenitor cells of AT2 organoids, consistent with our finding (Fig. 2). A TNFRSF12A^hi^ subset of basal cells were identified to be the progenitor cell of basal organoids, differentiating into ciliated cells and club cells after air-liquid interface culture or prolonged 3D culture. Further study is warranted to determine whether a similar fraction of cells are present in our lung organoids and serve a comparable role.

Alveolar organoids sustain productive SARS-CoV-2 infection (Fig. 4h). However, SARS-CoV-2 replicative fitness in alveolar organoids is lower than that in airway organoids (Figs. 4a and 5c), which might be ascribed to fewer ACE2^+^ cells in alveolar organoids compared to airway organoids (Fig. 3b). Consistently, an earlier study reported a decreasing gradient of ACE2 expression and SARS-CoV-2 infection in primary cells isolated from proximal and distal respiratory mucosa^9^. The higher replicative fitness in airway organoids than in alveolar organoids reveals the pathological basis of SARS-CoV-2 high transmissibility among humans. The 2D airway organoids cultured at pH 6.6/7.4, the physiological milieu of native airway epithelium, show a strengthened epithelial barrier and sustain a more productive SARS-CoV-2 infection than the original organoids cultured at pH 7.4/7.4 (Fig. 5c, d). The findings indicate that reconstituting a physiological milieu improves the ability of organoids to simulate in vivo tissues. Our results also demonstrated that the acidic pH promoted SARS-CoV-2 viral growth via the elevated ACE2 expression^52^. These optimized 2D airway organoids model the high infectivity of SARS-CoV-2 than most, if not all, reported organoid models and primary respiratory cells. Importantly, these 2D airway organoids recapitulate the Omicron variant’s higher infectivity and replicative fitness than an ancestral strain (Fig. 6a–d). Moreover, the higher replicative fitness of the Omicron variant only occurs in the airway organoids, not in alveolar organoids (Fig. 6e), which correlates with Omicron’s higher transmissibility and lower risk of developing a lung infection. In addition, both WT and the Omicron variant target ACCTUB^+^ ciliated cells in 2D airway organoids (Fig. 6d), consistent with the observations in cultured human airway epithelial cells and respiratory cells collected from COVID-19 patients^37,41,42^. Notably, a very distinct pattern of replicative fitness was observed in VeroE6/TMPRSS2 cells infected with the Omicron and WT viruses, which highlights the physiological relevance of the airway organoids for modeling infection (Supplementary Fig. S11).

Taken together, we establish a two-phase, bipotential organoid culture system that enables the generation of both types of human respiratory epithelium in culture plates with high efficiency. The lung organoids provide a stable and self-renewable source for long-term expansion, while terminally differentiated airway and alveolar organoids faithfully phenocopy the human airway and alveolar epithelium respectively. Alveolar organoids can be utilized to study the pathogenesis of SARS-CoV-2-related pneumonia. The optimized 2D airway organoids adequately recapitulate the high infectivity of SARS-CoV-2 and serve as a robust in vitro tool to determine the infectivity of SARS-CoV-2 variants in the human airways. Overall, these organoid models provide a unique and physiologically-active tool for broad applications to combat SARS-CoV-2 and other respiratory viruses.

## Materials and methods

### Establishment, maintenance, and differentiation of respiratory organoids

This study was approved by the Institutional Review Board of the University of Hong Kong/Hospital Authority Hong Kong West Cluster (UW13-364 and UW21-695). A total of nine lines of human lung organoids were established previously using lung tissues from nine patients who underwent surgical resection owing to various diseases^19,21^. We used normal lung tissues adjacent to the diseased tissues for organoid culture. These lung tissues typically contained bronchioles of variable size surrounded by alveolar sacs. During the initial culture, progenitor cells proliferated as a result of the niche factors supplemented in a well-defined expansion medium, whereas other unrelated cells, including stromal cells, undergo extensive cell death. After 1–2 passages that took 2 to 3 weeks, fibroblasts and other non-epithelial components in the initial culture disappeared gradually. Afterward, the lung organoids consisting of pure epithelial cells proliferated and were stably expanded in an expansion medium for at least 1 year. The lung organoids were passaged every 2 to 3 weeks with a ratio between 1:3 to 1:10, depending on whether mechanical shearing or enzymatic digestion was used to split the organoids. The proximal differentiation protocol for generating 2D and 3D airway organoids was described previously^19^.

To direct distal differentiation into alveolar organoids, after treatment with 10× TrypLE Select (Invitrogen) for 5 min at 37 °C, we cultured the single cells dissociated from the lung organoids in distal differentiation (DD) medium (advanced DMEM/F-12 supplemented with 1% HEPES, 1% GlutaMAX, 1% Penicillin/Streptomycin, 50 nM dexamethasone, 100 μM 8-bromo-cAMP, 100 μM IBMX, 2% B-27, 50% Wnt3a conditioned medium, and 100 μg/mL Primocin) in Nunclon Sphera super-low attachment plate (Thermo) at 37 °C in a humidified incubator with 5% CO_2_. Alternatively, a Wnt agonist CHIR99021 (Toris) was supplemented in the DD medium with a concentration of 3 µM as a replacement for the Wnt3a conditioned medium. The medium was replenished every other day for 12–14 days.

Undifferentiated lung organoids, airway organoids, and alveolar organoids were harvested and applied to RNA extraction using a MiniBEST Universal RNA Extraction kit (Takara), followed by reverse transcription using a Transcriptor First-Strand cDNA Synthesis Kit (Roche) and oligo(dT) primer. The resultant cDNAs were used to measure mRNA expression levels of cellular genes (Supplementary Table S1) using the LightCycler 480 SYBR Green I Master Mix (Roche). Photomicrographs of the organoids were acquired using the Nikon Eclipse TS100 Inverted Routine Microscope.

### Virus infection and detection

SARS-CoV-2 isolate HKU-001a (WT, GenBank accession number MT230904) and an Omicron variant (B.1.1.529; GenBank OM212473) were previously reported^53,54^. These viruses were propagated in VeroE6/TMPRSS2 cells (JCRB1819) purchased from JCRB Cell Bank and titrated with plaque assay as we described previously^19^. 3D airway organoids were sheared mechanically and incubated with the virus for 2 h at 37 °C. The inoculated organoids were re-embedded into Matrigel and then incubated in basal medium (advanced DMEM/F-12 (Gibco) supplemented with 1% HEPES, 1% GlutaMAX, and 1% Penicillin/Streptomycin). After washing twice with the basal medium, 2D human airway organoids were apically incubated with the virus for 2 h at 37 °C, followed by incubation in the basal medium. The differentiated human alveolar organoids were incubated with the cultured virus for 2 h at 37 °C, followed by incubation in the basal medium. To assess replication kinetics, at the indicated hours after an MOI of 0.1 inoculation in 2D organoids and an MOI of 1 inoculation in 3D organoids, we harvested cell-free culture media, followed by RNA extraction using the MiniBEST Viral RNA/DNA Extraction Kit (Takara) and detection of viral loads (viral gene copy number of RdRp gene) by one-step RT-qPCR assay, and viral titration by TCID_50_ assay as described previously^55^. All experiments with live viruses were conducted in biosafety level 3 laboratories after approval by the Faculty of Medicine, The University of Hong Kong.

### Immunofluorescence staining and flow cytometry

The cellular composition of the organoids was characterized using specific antibodies (Supplementary Table S2) to recognize AT1 (AQP5), AT2 (SFTPB, SFTPC, and HTII-280), ciliated (ACCTUB and FOXJ1), goblet (MUC5AC), club (CC10), and basal cells (P63 and CK5), followed by secondary antibodies. Cellular proteins including ACE2 and TMPRSS2 in the organoids were labeled with corresponding antibodies. Z-stack scanning was performed on 2D airway organoids. The orthogonal projection was applied to generate cross-sectional images. To identify virus-infected cells, we stained virus-inoculated or mock-infected organoids using an in-house antibody against SARS-CoV-2 nucleoprotein (NP) raised in a guinea pig, after fixation with 4% paraformaldehyde (PFA) for 1 h at room temperature, permeabilization with 0.5% Triton X-100 for 10 min and blocking with protein block (Dako) for 1 h. To define cellular tropism, we co-stained the infected organoids with the α-NP and cell-type antibodies. Nuclei and actin filaments were counterstained with DAPI (Thermo Fisher Scientific) and Phalloidin-647 (Sigma-Aldrich), respectively. We whole-mounted the organoids on a glass slide with ProLong™ Glass Antifade Mountant (Invitrogen) after staining. Confocal images were acquired using a Carl Zeiss LSM 800 confocal microscope. Image processing was done with the ZEN blue software.

For flow cytometry analysis, organoids were dissociated into single cells with 10 mM EDTA (Invitrogen) for 30–60 min at 37 °C, fixed with 4% PFA for 15 min at room temperature, permeabilized with 0.1% Triton X-100 for 5 min at 4 °C, and then applied to immunostaining using the specific antibodies (Supplementary Table S2) and the corresponding isotype controls were used for gating. To determine infection rate, after an inoculation at an MOI of 10, organoids were dissociated into single cells, fixed with 4% PFA, followed by immunostaining using an α-double-stranded RNA antibody (dsRNA, 10010500, Scicons); mock-infected organoids were used for gating. BD FACSCantoII Analyzer or LSR Fortessa was used for analysis. FlowJo software was used for data processing.

### Fluorescence-activated cell sorting

Single cells dissociated from lung organoids were labeled with 100 nM LysoTracker™ Red DND-99 (L7528, Invitrogen) or an anti-HTII-280 (TB-27AHT2-280, Terrace Biotech) in staining buffer (phosphate-buffered saline (PBS) with 2% fetal bovine serum (FBS), 1 mM EDTA, 200 µg/mL DNase, and 10 µM Y-27632). The cells were then re-suspended in sorting buffer (DMEM no phenol red with 1% HEPES, 1% GlutaMAX, 1% Penicillin/Streptomycin, and 10 µM Y-27632) for isolating AT2 cells using a BD FACSAria Fusion Cell Sorter. The cells harvested in collection buffer (basal medium with 20% FBS and 10 µM Y-27632) were then embedded in Matrigel overlaid with expansion medium or suspension-cultured with DD medium.

### Live-cell imaging to assess AT2 cell functionality in alveolar organoids

Differentiated human alveolar organoids were incubated with 1 µM β-BODIPY™ FL C12-HPC (D3792, Invitrogen) in a DD medium for 24 h at 37 °C. After washing with basal medium, the organoids were incubated with 100 nM LysoTracker™ Red DND-99 (L7528, Invitrogen) in DD medium for 30 min at room temperature. The organoids were then counterstained with Hoechst 33342 (62249, Thermo) and CellMask™ Deep Red plasma membrane stain (C10046, Invitrogen) and applied to confocal imaging using a Carl Zeiss LSM 800 confocal microscope.

### RNA sequencing analysis

Human lung organoids were induced differentiation to generate 3D airway organoids, 2D airway organoids, and 3D alveolar organoids in triplicate as described above or previously^19^. Three biological replicates of lung organoids and derived organoids were harvested for RNA extraction using an RNAeasy Mini Kit (Qiagen). After a quality check with Bioanalyzer, RNA specimens were applied to library preparation using a KAPA mRNA HyperPrep Kit. In brief, Poly-A-containing mRNA was collected by using poly-T oligo-attached magnetic beads. The purified mRNA was fragmented to 200–300 bp by incubating at 94 °C for 6 min in the presence of magnesium ions. The fragmented mRNA was then applied as a template to synthesize the first-strand cDNA by using random hexamer primer and reverse transcriptase. In the second strand cDNA synthesis, the mRNA template was removed and a replacement strand was generated to form the blunt-end double-stranded (ds) cDNA. The ds cDNA underwent 3′ adenylation and indexed adapter ligation. The adapter-ligated libraries were enriched by 15 cycles of polymerase chain reaction (PCR). The libraries were denatured and diluted to optimal concentration. Illumina NovaSeq 6000 was used for 151 bp Pair-End sequencing. Sequencing reads were assigned to individual samples. Each sample had an average throughput of 9.6 Gb and a total throughput of 111.5 Gb. In terms of sequence quality, an average of 93% of the bases achieved a quality score of Q30 where Q30 denotes the accuracy of a base call to be 99.9%. Sequencing reads were mapped to the human reference genome GRCh38 with the RNA-Seq aligner STAR. Clean reads were quantified based on the number of reads spanning the back-splicing junction, and their fragments per kb for a million reads (FPKM) were calculated using HTSeq 0.13.5. Aligned BAM files were sorted by SAMtools. GenomicAlignments and DESeq2 R packages (version 1.18.1) were used for read counting and differential expression analysis. Genes with an absolute value of log_2_ (fold change) greater than 1 and adjusted *P* value (Benjamini–Hochberg) less than 0.05 were considered to be differentially expressed. The RNA-seq data were uploaded to the GEO database with an accession number GSE189514 (token: qdwnkcagnnyrrgt)

### Transmission electron microscopy

Alveolar organoids, airway organoids, and airway organoids infected by SARS-CoV-2 (MOI = 5) were embedded in resin after sequential fixation in 2.5% glutaraldehyde and 1% osmium. The ultrathin sections were stained with uranyl acetate and examined under an FEI Tecnai G2 20 S-TWIN scanning transmission electron microscope.

### Optimization of 2D airway organoids

2D airway organoids were generated as described previously^19^. One day after seeding and incubation with the expansion medium, cells became 60%–90% confluent on the transwell membrane. We then replaced the expansion medium with a proximal differentiation medium to induce proximal differentiation, and the cells reached 100% confluence in 1–2 days. Subsequently, the proximal differentiation media in the bottom chambers were buffered with HEPES (Gibco) to create a pH of around 7.4, while those in the top chambers were buffered with HEPES or PIPES (Sigma-Aldrich), the latter maintained a pH of around 6.6. Organoids were then incubated in these proximal differentiation media for 10–12 days to achieve maturation.

### Statistical analysis

Statistical analysis was conducted using GraphPad Prism 9.0. Either Student’s *t*-test or ANOVA test was used to determine statistical significance as specified in the figure legends. The number of replicates is indicated in the figure legends. *P ≤ 0.05, **P ≤ 0.01, and ***P ≤ 0.001.

## Supplementary information

Supplementary Information
