# Supplementary material for: A bipotential organoid model of respiratory epithelium recapitulates high infectivity of SARS-CoV-2 Omicron variant
Source: Cell Discov. 2022 Jun 17;8:57. doi: 10.1038/s41421-022-00422-1 (PMC9203776; doi:10.1038/s41421-022-00422-1)
Supplement: Supplementary file 1 — Supplementary Information [file 41421_2022_422_MOESM1_ESM.pdf]

# A bipotential organoid model of respiratory epithelium recapitulates high infectivity of SARS-CoV-2 Omicron variant

## SUPPLEMENTARY INFORMATION

**Supplementary Fig. S1**. Cell populations in lung organoids.

Lung organoids were applied to flow cytometry to examine the percentage of airway and alveolar epithelial cell types. (a) Representative histograms of the cell-type markers. (b) Data represent the means ± SD of duplicated organoid samples from one donor.

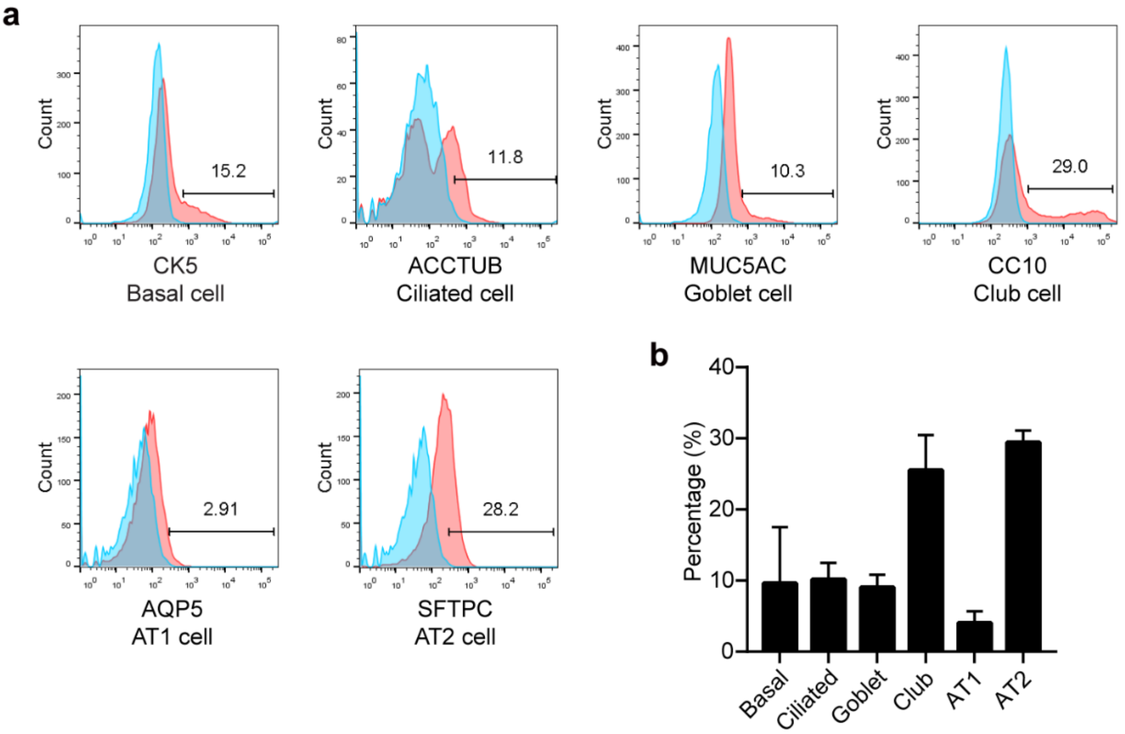

**Supplementary Fig. S2.** Marker gene expression in lung organoids during long-term culture.

Normalized expression levels of airway and alveolar cellular markers in expanding lung organoids were measured over the indicated time course. Data represent the means ± SD of duplicated organoid samples from one donor.

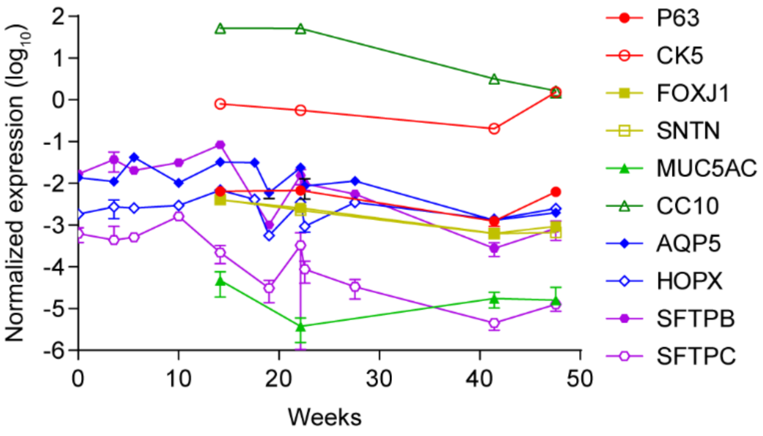

**Supplementary Fig. S3**. Reproducible alveolar differentiation in two lines of lung organoids.

Normalized expression levels of AT1- and AT2-cell markers were assessed in parental lung organoids (LO) from two different donors (a, b) and their derived alveolar organoids (AlvO). Data represent the means ± SD of a representative experiment in each organoid line, $n = 2$. Two-tailed unpaired Student's t-test.

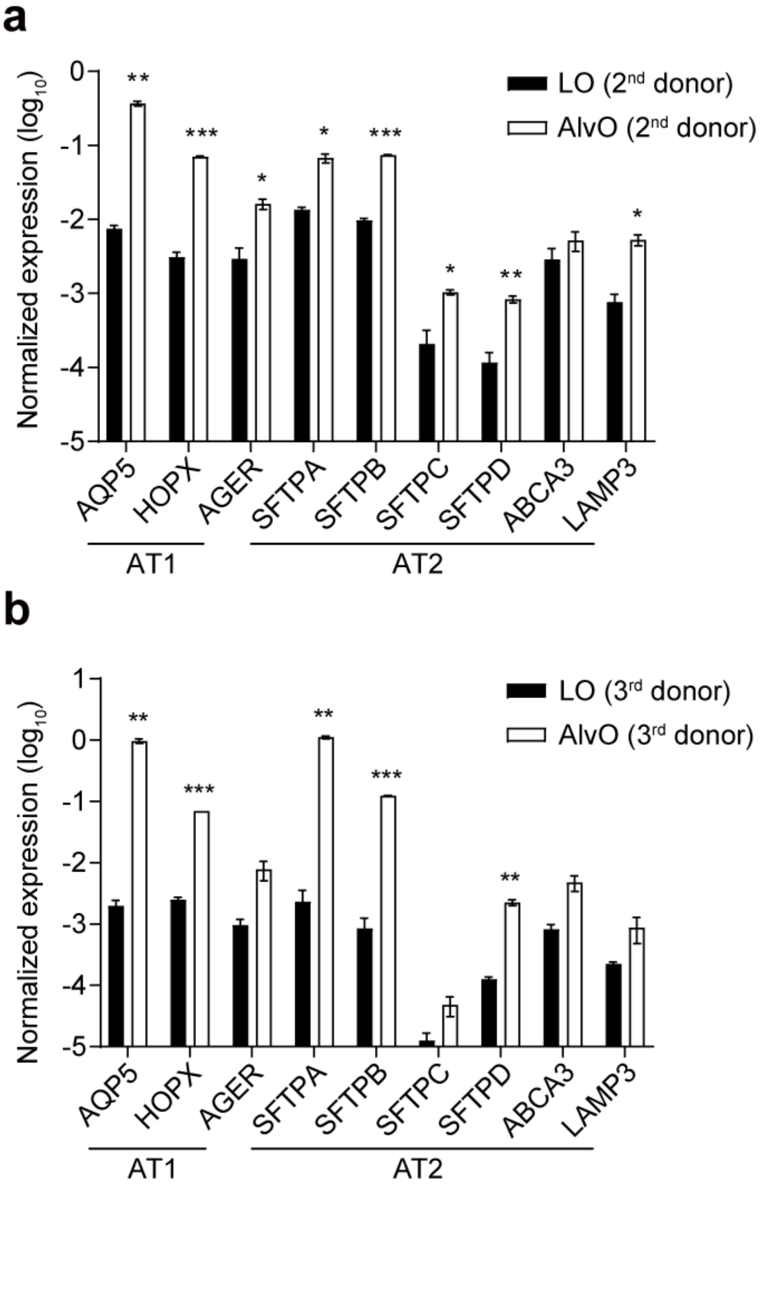

**Supplementary Fig. S4**. Suspension culture is required to induce alveolar differentiation.

Lung organoids were maintained in Matrigel overlaid with the expansion medium (black) or single cells suspension-cultured in DD medium (grey) or single cells embedded in Matrigel overlaid with DD medium (white) for 2 weeks. (a) Photomicrographs present the organoid morphology of day 14 after single cells were suspension-cultured in DD medium or embedded in Matrigel overlaid with DD medium. Scale bar, 100 μm. (b) The RT-PCR results show normalized expression levels of AT1- and AT2-cell markers. Data represent the means ± SD of a representative experiment, $n = 2$. Two-tailed unpaired Student's t-test.

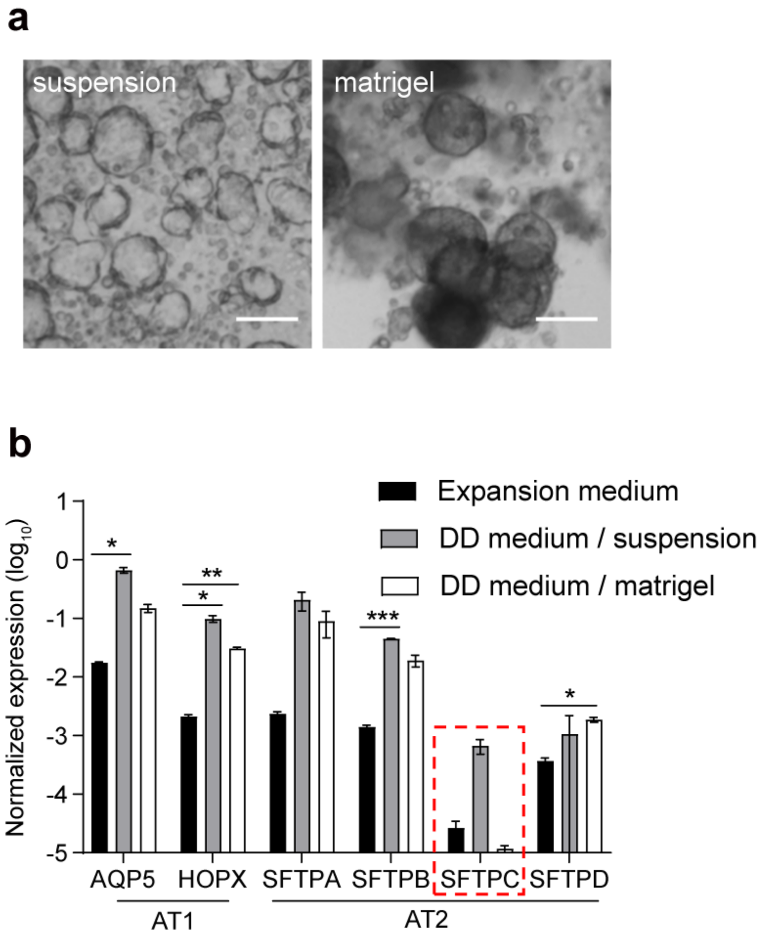

**Supplementary Fig. S5.** A WNT agonist induces alveolar differentiation.

Lung organoids were 3D cultured in the expansion medium (black) or suspension-cultured with DD medium supplemented with either Wnt3a conditioned medium (grey) or CHIR99021 (white) for 2 weeks and then applied to RT-qPCR assay. The results show normalized expression levels of AT1- and AT2-cell markers. Data represent the means ± SD of a representative experiment, *n* = 3. Two-tailed unpaired Student's t-test.

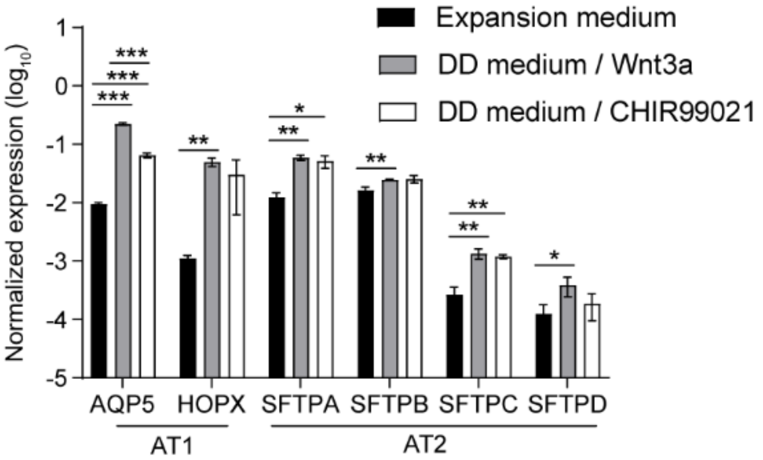

**Supplementary Fig. S6**. Reproducible derivation of alveolar organoids from lung organoids during 6 consecutive passages.

Normalized expression levels of AT1- (AQP5, HOPX) and AT2- (SFTPA, SFTPB, SFTPC, SFTPD) cell markers were assessed pairwise in lung organoids (LO) from one donor and the derived alveolar organoids (AlvO) during six consecutive passages. Data represent the means ± SD of an experiment, $n = 2$. Two-tailed unpaired Student's t-test.

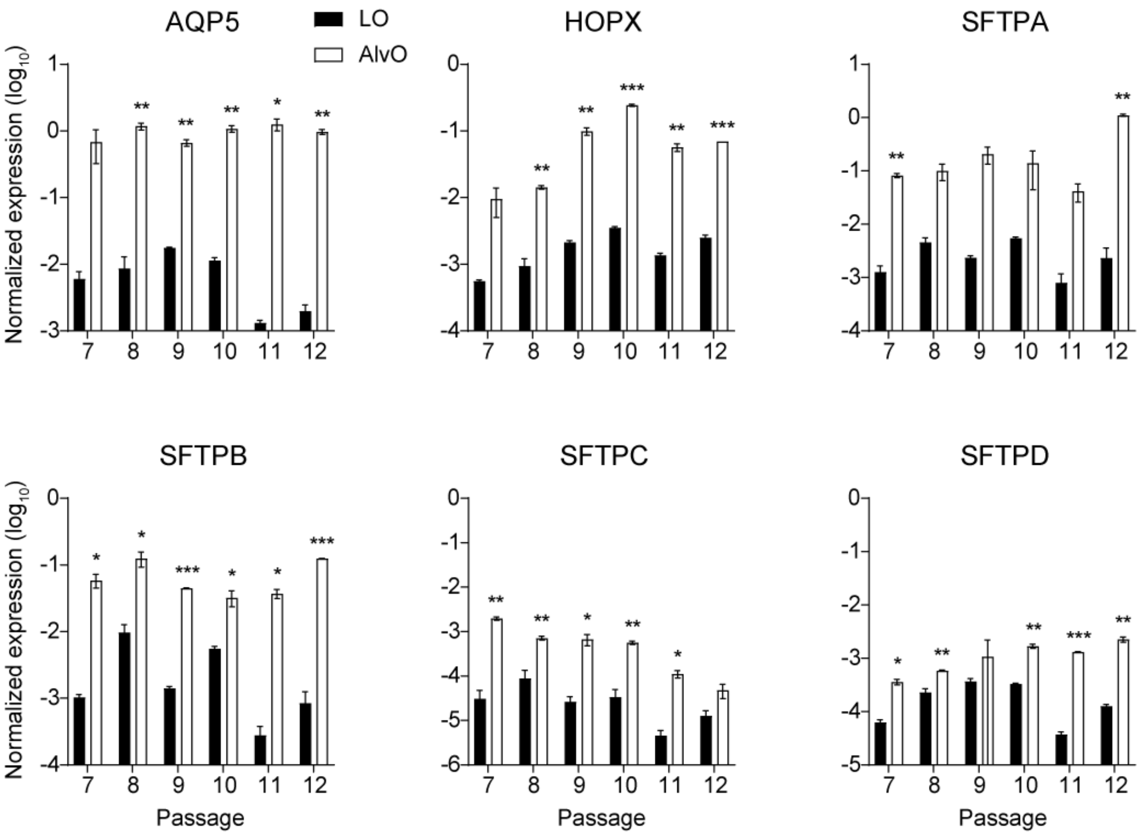

**Supplementary Fig. S7**. Higher abundance of cell-type proteins in alveolar organoids.

Parental LOs and differentiated AlvOs were applied to flow cytometry to examine the percentage (shown in Fig. 1D) and the intensity of $AQP5^+$ AT1 and $SFTPC^+$/LysoTracker $red^+$/$HTII\text{-}280^+$ AT2 cells. Data represent the means ± SD of the mean fluorescent intensity (MFI) of the positive cells in a representative experiment in one organoid line, *n* = 3 for AQP5 and SFTPC, *n* = 2 for LysoTracker red and HTII-280. Two-tailed unpaired Student's t-test.

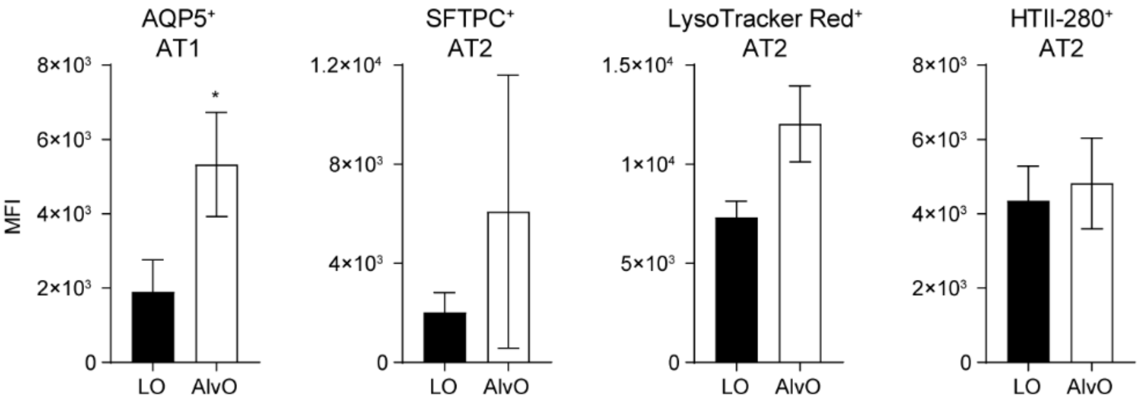

**Supplementary Fig. S8**. Staining and gating strategy for sorting AT2 cells in lung organoids.

(a) Top, histograms show the fluorescent signals of LysoTracker red (left, PE) and HTII-280 (right, FITC) of the stained lung organoids gated by isotype controls. Red, cells stained with the dye or the specific antibody; blue, cells mock-stained or stained with an isotype control. Bottom, dot plots show the stepwise gating of the single-cell suspension from lung organoids. (b) Lung organoids derived from sorted AT2 cells (AT2-LO) were applied to flow cytometry to examine the percentage of airway and alveolar epithelial cell types. Representative histograms of the cell-type markers were shown.

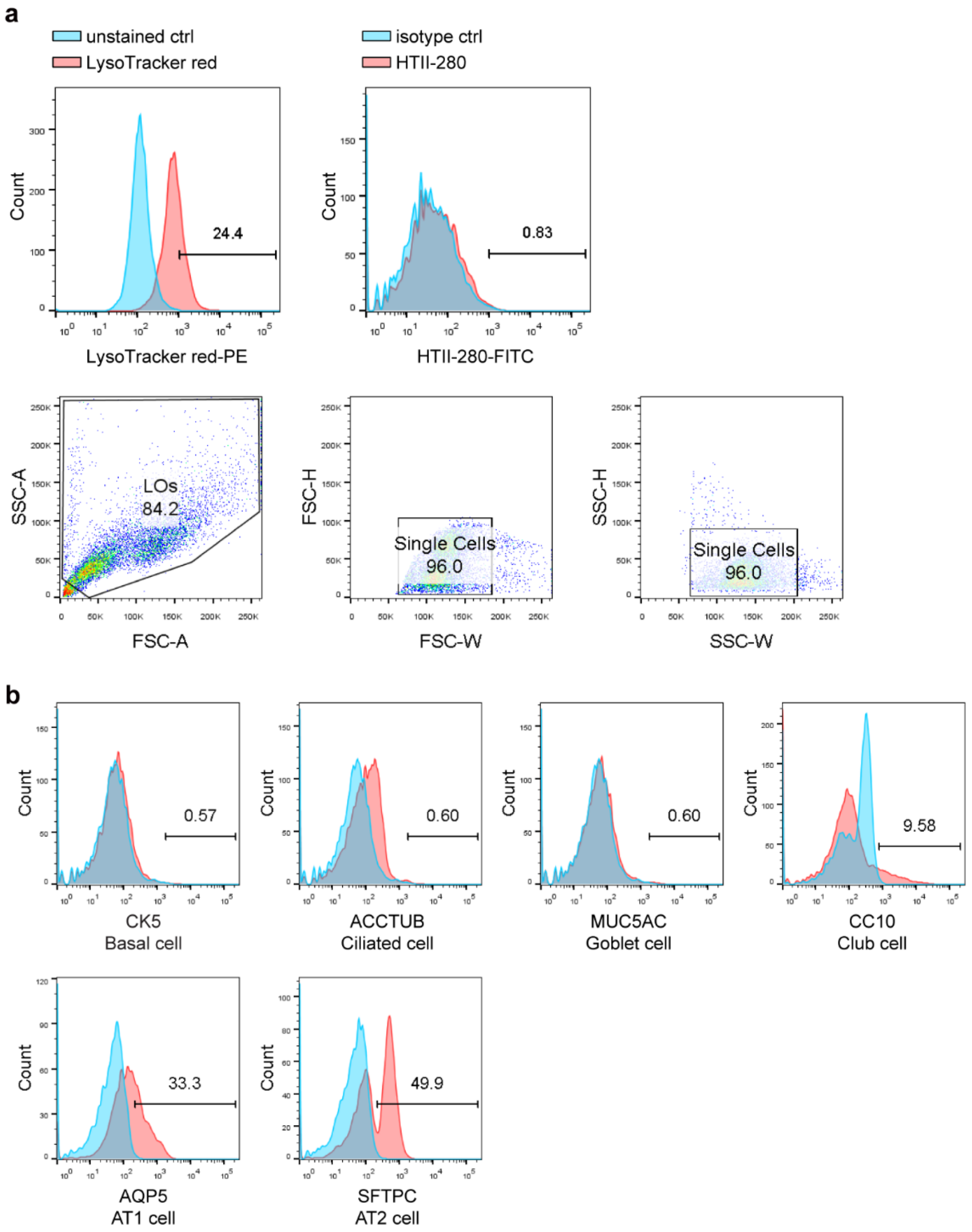

**Supplementary Fig. S9**. ACE2 expression in ciliated cells and AT2 cells.

ACE2 and TMPRSS2 expression in airway organoids (3D AwO & 2D AwO) and alveolar organoids (AlvO) differentiated from the same organoid line were detected by flow cytometry. Representative dot plots show the AwOs co-stained with ACCTUB (PE-Texas red) and ACE2 (FITC), and the AlvOs co-stained with SFTPC (PE-Texas red) and ACE2 (FITC). Red, cells stained with specific antibodies; blue, cells stained with isotype controls.

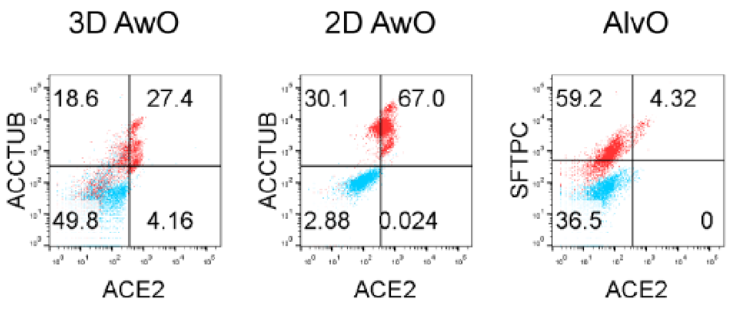

**Supplementary Fig. S10**. Increased ciliated cell population and ACE2 expression in 2D airway organoids cultured in slightly acidic medium.

Flow cytometry was performed to analyze the percentages of (a) ACCTUB$^+$ ciliated cells, (b) ACE2$^+$ and TMPRSS2$^+$ cells in the 2D AwOs differentiated in pH 6.6/7.4 or 7.4/7.4. Data represent the means ± SD of a representative experiment, *n* = 4 (ACCTUB) or *n* = 2 (ACE2/TMPRSS2) respectively. Two-tailed unpaired Student's t-test.

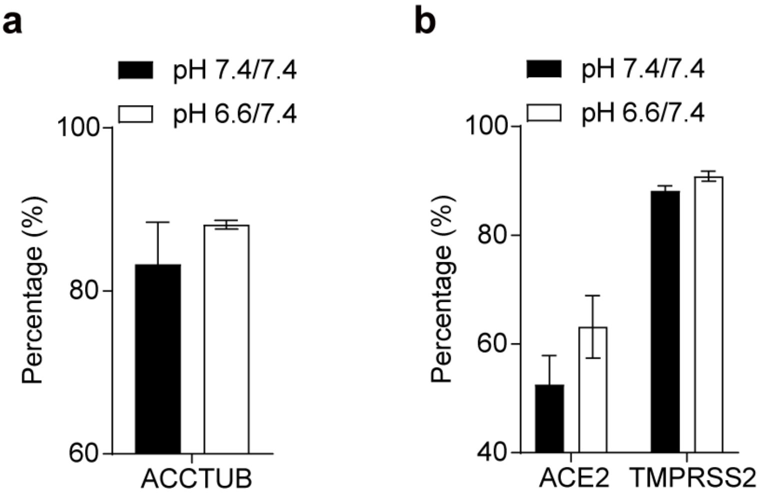

**Supplementary Fig. S11**. Replicative fitness of SARS-CoV-2 variants in Vero E6/TMPRSS2 cells.

At the indicated hours after inoculation with wildtype (WT) or the Omicron variant (MOI = 0.01), culture media were harvested from the VeroE6/TMPRSS2 cells and applied to viral load detection and viral titration by TCID50 assay. Data show means ± SD of a representative experiment, $n$ = 3. Two-tailed unpaired Student's t-test.

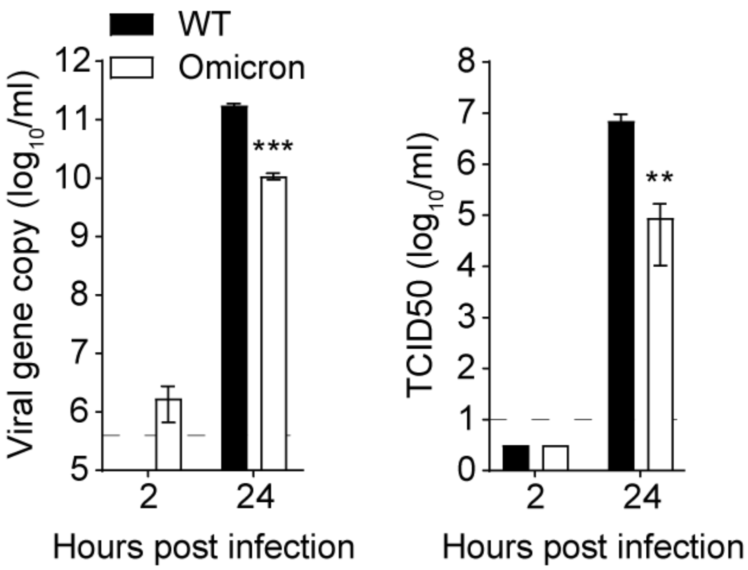

**Supplementary Table S1**. qPCR Primer list.

| **Gene** | **Sequence** | |
|---|---|---|
| **SARS-CoV-2 RdRp** | F | CGCATACAGTCTTRCAGGCT |
| | R | GTGTGATGTTGAWATGACATGGTC |
| | Probe | FAM-TTAAGATGTGGTGCTTGCATACGTAGAC-lABkFQ |
| **AQP5** | F | GCTCACTGGGTTTTCTGGGTA |
| | R | TCCATGGTCTTCTTCCGCTC |
| **HOPX** | F | GCCTTTCCGAGGAGGAGAC |
| | R | TCTGTGACGGATCTGCACTC |
| **AGER** | F | GTGTCCTTCCCAACGGCTC |
| | R | ATTGCCTGGCACCGGAAAA |
| **SFTPA** | F | GATGGGCAGTGGAATGACAGG |
| | R | GGAATGAAGTGGCTAAGGGT |
| **SFTPB** | F | GCAGACGTCCATAGCTCCTC |
| | R | AGGGACACTTCCAGGCATTT |
| **SFTPC** | F | AGCAAAGAGGTCCTGATGGA |
| | R | CGATAAGAAGGCGTTTCAGG |
| **SFTPD** | F | TGGGCTTCCAGATGTTGCTT |
| | R | CGACACTTTGGCCATTTGGG |
| **ABCA3** | F | AGATGTAGCGGACGAGAGGA |
| | R | GCTGCTCGTACACCTTGGAG |
| **LAMP3** | F | AAGATGACCACTTTGGAAATGTG |
| | R | GATGGCCCCAATCACAGGAA |
| **P63** | F | CAGACTCAATTTAGTGAGCC |
| | R | CTGCTGGTCCATGCTGTT |
| **CK5** | F | GAGGAATGCAGACTCAGTGGA |
| | R | TAGCTTCCACTGCTACCTCCG |
| **FOXJ1** | F | TCGTATGCCACGCTCATCTG |
| | R | CGGATTGAATTCTGCCAGGT |
| **SNTN** | F | GCTGCAAACCCAATTTAGGA |
| | R | TGCTCATCAAGTTCAGAAAGGA |
| **MUC5AC** | F | CCTACAAAGCTGAGGCCTGT |
| | R | GACCCTCCTCTCAATGGTGC |
| **CC10** | F | AGCATCATTAAGCTCATGGAAAAA |
| | R | GTGGACTCAAAGCATGGCAG |
| **GAPDH** | F | GGAGCGAGATCCCTCCAAAAT |
| | R | GGCTGTTGTCATACTTCTCATGG |

**Supplementary Table S2**. Antibody list.

| Name | Supplier | Cat. no. | Lot no. | Dilution (IF) | Dilution (Flow) |
|---|---|---|---|---|---|
| **Mouse IgG1** | abcam | ab91353 | GR3327311-3 | - | 1:100 |
| **Mouse IgM** | abcam | ab18401 | GR3336129-2 | - | 1:100 |
| **Rabbit IgG** | abcam | ab172730 | GR3284310-8 | - | 1:100 |
| **Goat IgG** | R&D systems | AB-108-C | ES4120071 | - | 1:100 |
| **Rat IgG** | invitrogen | 10700 | RA226537 | - | 1:100 |
| **ACE2** | R&D systems | AF933 | HOK0620051 | 1:100 | 1:100 |
| **TMPRSS2** | invitrogen | PA5-14264 | SI2433683G | - | 1:100 |
| **AQP5** | abcam | ab92320 | GR97210-39 | 1:100 | 1:100 |
| **SPB** | Santa Cruz Biotech | sc-133143 | D2418 | 1:50 | - |
| **pro-SPC** | Sigma-Aldrich | AB3786 | 3041829 | - | 1:100 |
| **HTII-280** | Terrace Biotech | TB-27AHT2-280 | KM27 | 1:100 | 1:100 |
| **P63** | abcam | ab124762 | GR303296-1 | 1:100 | - |
| **CK5** | abcam | ab128190 | GR239416-18 | - | 1:100 |
| **FOXJ1** | Sigma-Aldrich | HPA005714 | G114396 | 1:50 | - |
| **TUBULIN** | abcam | ab179509 | GR252919-6 | - | 1:1000 |
| **TUBULIN** | Sigma-Aldrich | T7941 | 088M4793 | 1:100 | 1:1000 |
| **CC10** | R&D systems | MAB4218 | YWN0218091 | - | 1:100 |
| **MUC5AC** | abcam | ab3649 | GR126783 | - | 1:1000 |
| **SARS-CoV-2 NP** | in-house | - | - | 1:1000 | - |
| **J2 (dsRNA)** | Scicons | 10010500 | 17741 | - | 1:100 |
| **Goat anti-Mouse 488** | invitrogen | A-11001 | 2090562 | 1:500 | 1:500 |
| **Goat anti-Mouse 594** | invitrogen | A-11005 | 1750828 | 1:500 | - |
| **Goat anti-Rabbit 488** | invitrogen | A-11034 | 1885241 | 1:500 | 1:500 |
| **Goat anti-Rabbit 594** | invitrogen | A-11037 | 1608397 | 1:500 | - |
| **Donkey anti-Goat 488** | invitrogen | A-11055 | 1771339 | 1:500 | 1:500 |
| **Donkey anti-Rat 488** | invitrogen | A-21208 | 1900239 | - | 1:500 |
| **Goat anti-Guinea pig 488** | invitrogen | A-11073 | 1990462 | 1:500 | - |
